# A targeted genomic alteration analysis predicts survival of melanoma patients under BRAF inhibitors

**DOI:** 10.18632/oncotarget.26707

**Published:** 2019-03-01

**Authors:** Baptiste Louveau, Julie Delyon, Coralie Reger De Moura, Maxime Battistella, Fanelie Jouenne, Lisa Golmard, Aurelie Sadoux, Marie-Pierre Podgorniak, Ichrak Chami, Oren Marco, Julie Caramel, Stephane Dalle, Jean-Paul Feugeas, Nicolas Dumaz, Celeste Lebbe, Samia Mourah

**Affiliations:** ^1^ Paris-Diderot University, Sorbonne Paris Cité, Paris, France; ^2^ Paris-Diderot University, Inserm, UMR_S976, Paris, France; ^3^ Department of Pharmacogenomics, Saint-Louis Hospital, AP-HP, Paris, France; ^4^ Department of Dermatology, Saint-Louis Hospital, AP-HP, Paris, France; ^5^ Department of Pathology, Saint-Louis Hospital, AP-HP, Paris, France; ^6^ Paris Diderot University, Inserm, UMR_S1165, Paris, France; ^7^ Department of Genetics, Pôle de Médecine Diagnostique et Théranostique, Institut Curie, Paris, France; ^8^ Department of Plastic, Reconstructive and Esthetic Surgery, Saint-Louis Hospital, AP-HP, Paris, France; ^9^ Université de Lyon, Université Claude Bernard Lyon 1, INSERM 1052, CNRS 5286, Centre Léon Bérard, Cancer Research Center of Lyon, Equipe Labellisée Ligue contre le Cancer, Lyon, France; ^10^ Centre Hospitalier Lyon Sud, Hospices Civils de Lyon, Pierre Bénite, France; ^11^ Université de Franche-Comté, Inserm, UMR_1137, Paris, France

**Keywords:** melanoma, BRAF inhibitors, targeted genomic alteration, predictive analysis, targeted therapy resistance

## Abstract

Several mechanisms have been described to elucidate the emergence of resistance to MAPK inhibitors in melanoma and there is a crucial need for biomarkers to identify patients who are likely to achieve a better and long-lasting response to BRAF inhibitors therapy. In this study, we developed a targeted approach combining both mRNA and DNA alterations analysis focusing on relevant gene alterations involved in acquired BRAF inhibitor resistance. We collected baseline tumor samples from 64 melanoma patients at BRAF inhibitor treatment initiation and showed that the presence, prior to treatment, of mRNA over-expression of genes’ subset was significantly associated with improved progression free survival and overall survival. The presence of DNA alterations was in favor of better overall survival. The genomic analysis of relapsed-matched tumor samples from 20 patients allowed us to uncover the largest landscape of resistance mechanisms reported to date as at least one resistance mechanism was identified for each patient studied. Alterations in *RB1* have been most frequent and hence represent an important additional acquired resistance mechanism. Our targeted genomic analysis emerges as a relevant tool in clinical practice to identify those patients who are more likely to achieve durable response to targeted therapies and to exhaustively describe the spectrum of resistance mechanisms. Our approach can be adapted to new targeted therapies by including newly identified genetic alterations.

## INTRODUCTION

In the last few years, therapies targeting the mitogen activated-protein kinase (MAPK) pathway have significantly extended progression free survival (PFS) and overall survival (OS) in patients with BRAFV600 mutated metastatic melanoma compared to chemotherapy [1–3].

BRAFV600 mutations (*BRAF^V600mut^*) are detected in about 50% of lesions from metastatic melanoma patients and result in the constitutive activation of the MAPK pathway [4]. First used as a monotherapy, BRAF inhibitors, vemurafenib (Zelboraf^®^) and dabrafenib (Tafinlar^®^), have undergone multiple resistance mechanisms and their association with MEK inhibitors has become the standard of care [5–7] in order to reduce the occurrence of resistance.

In this context, the identification of biomarkers enabling a better understanding of the mechanisms of MAPK inhibitor resistance constitutes a great challenge to stratify subsets of patients more likely to achieve long lasting responses and hence to predict clinical benefit of such targeted therapies.

Studies on the mechanisms of acquired resistance to BRAF inhibitors, although conducted in relatively small cohorts, described, using DNA sequencing (Whole exome sequencing, WES), genetic alterations activating the MAPK pathway such as *NRAS* or *MAP2K1* activating mutations and *BRAF^V600E/K^* amplification in relapsed tumors [8–10]. Activation of the PI3K/AKT pathway consecutive to *PTEN* loss and alterations of genes involved in cell cycle such as *CDKN2A* as well as the formation of eIF4F complex have also been identified as resistance mechanisms [10–14].

In addition, studies focusing on targeted mRNA analysis have associated *BRAF* aberrant splice variants [10, 15] and gene expression alterations, namely overexpression of *MAP3K8/COT*, *IGF1* or other tyrosine kinase receptor encoding genes, to resistance [8, 16–18]. More recent studies, using WES, highlighted the existence of multiple resistance gene alterations within the same tumor [15, 19]. However, these studies using larger cohorts (*n* = 30 and 45) have shown that resistance to BRAF inhibitors remained unexplained for nearly half of the analyzed melanomas [15, 19, 20].

Considering the complexity of these mechanisms and the multiplicity of genes implicated in resistance to BRAF inhibitors, we aimed, in the present study, to demonstrate the clinical relevance of an innovative tool combining mRNA expression, copy number and mutation analyses of genes involved in the RAF/MEK pathway inhibition resistance in order to (i) identify patients who are more likely to achieve durable response to BRAF inhibitors and to (ii) provide an exhaustive landscape of acquired resistance mechanisms at relapse.

## RESULTS

**Figure 1:**
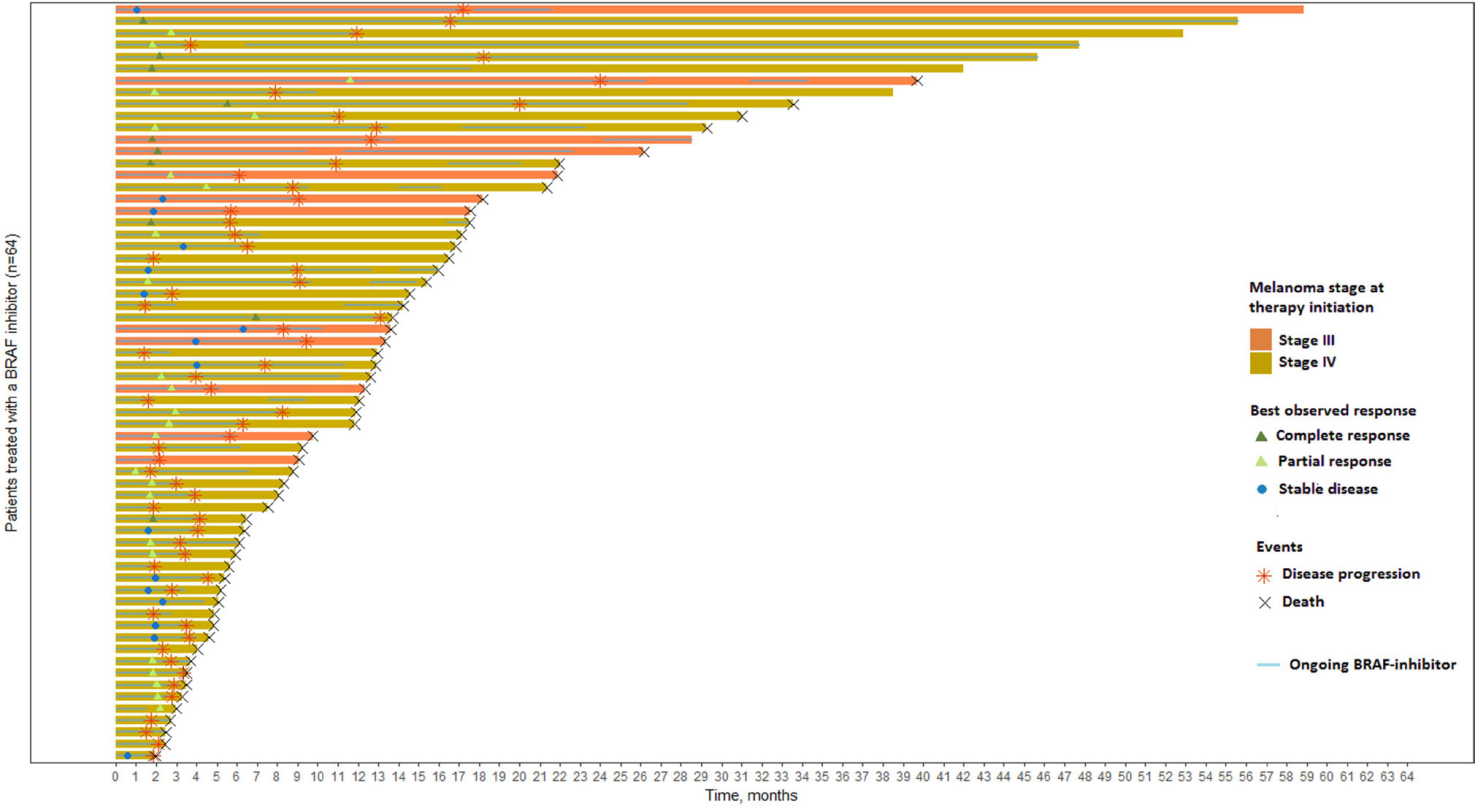
Swimmer plot of the 64 patients included and ranked according to their overall survival Patients are censored at last available date of follow-up if disease progression or death did not occur. T0 is the time of BRAF inhibitor initiation.

Of the 64 patients included in this study, 94 *BRAF^V600^* mutated tumor samples were collected; 64 were baseline samples (1 per patient), 20 were relapsed-matched samples and 10 corresponded to collection of multiple lesions at relapse. Table 1 summarizes the clinical and pathological characteristics at baseline and during the follow-up. Of the 64 patients, 12 (18.8%) presented an unresectable stage III and 52 (81.2%) a stage IV melanoma. Brain metastases were observed at baseline for 19 (29.7%) patients, underlining the clinical severity of our cohort. Fifty-nine (92.2%) patients received vemurafenib monotherapy as first line BRAF inhibitor treatment and 5 (7.8%) received dabrafenib. Under BRAF inhibitor treatment, a disease progression occurred in 60 (93.8%) patients with a median PFS of 4.5 months and death in 56 (87.5%) patients with a median OS of 12.6 months. A swimmer plot presents the clinical course and events of interest occurring during the follow-up of the 64 included patients (Figure 1).

**Table 1:** Clinical and pathological characteristics of included patients

	All included patients (*N* = 64)
**Age at therapy initiation, years**	57.3 ± 14.1 (57.0)
**Male sex**	39 (61.0%)
**Melanoma subtype**	
Nodular	18 (28.1%)
Superficial spreading melanoma	35 (54.7%)
Others	5 (7.8%)
Undetermined	6 (9.4%)
**Breslow thickness, mm**	3.2 ± 2.6 (2.4)
**Ulceration**	21 (41.1%)
**Stage**	
III unresectable	12 (18.8%)
IVa	6 (9.4%)
IVb	6 (9.4%)
IVc	40 (62.5%)
**Brain metastasis**	19 (29.7%)
**First BRAF inhibitor initiated**	
Vemurafenib	59 (92.2%)
Dabrafenib	5 (7.8%)
**Occurrence of a disease progression (Any time during the follow-up)**	60 (93.8%)
**Best observed clinical response**	
Complete remission	10 (15.6%)
Partial remission	25 (39.1%)
Stable disease	16 (25.0%)
Disease progression	13 (20.3%)

Data are mean ± sd (median) and number (%). Disease progression and best observed clinical response were assessed using RECIST v1.1.(mm: millimeters).

Univariate analysis of clinical baseline characteristics for PFS and OS are presented in Supplementary Table 1. For PFS, sex, melanoma stage, presence of brain metastasis and ulceration of the primary melanoma were candidate for Cox multivariate analysis. For OS, sex, melanoma subtype, melanoma stage, presence of brain metastasis, ulcerations and first BRAF inhibitor initiated were candidate variables. Proportional hazards assumption was tested with the scaled Schoenfeld residuals and was not rejected for these candidate variables.

### Tumor DNA alterations prior to treatment is in favor of better OS in melanoma patients treated with BRAF inhibitors

**Figure 2:**
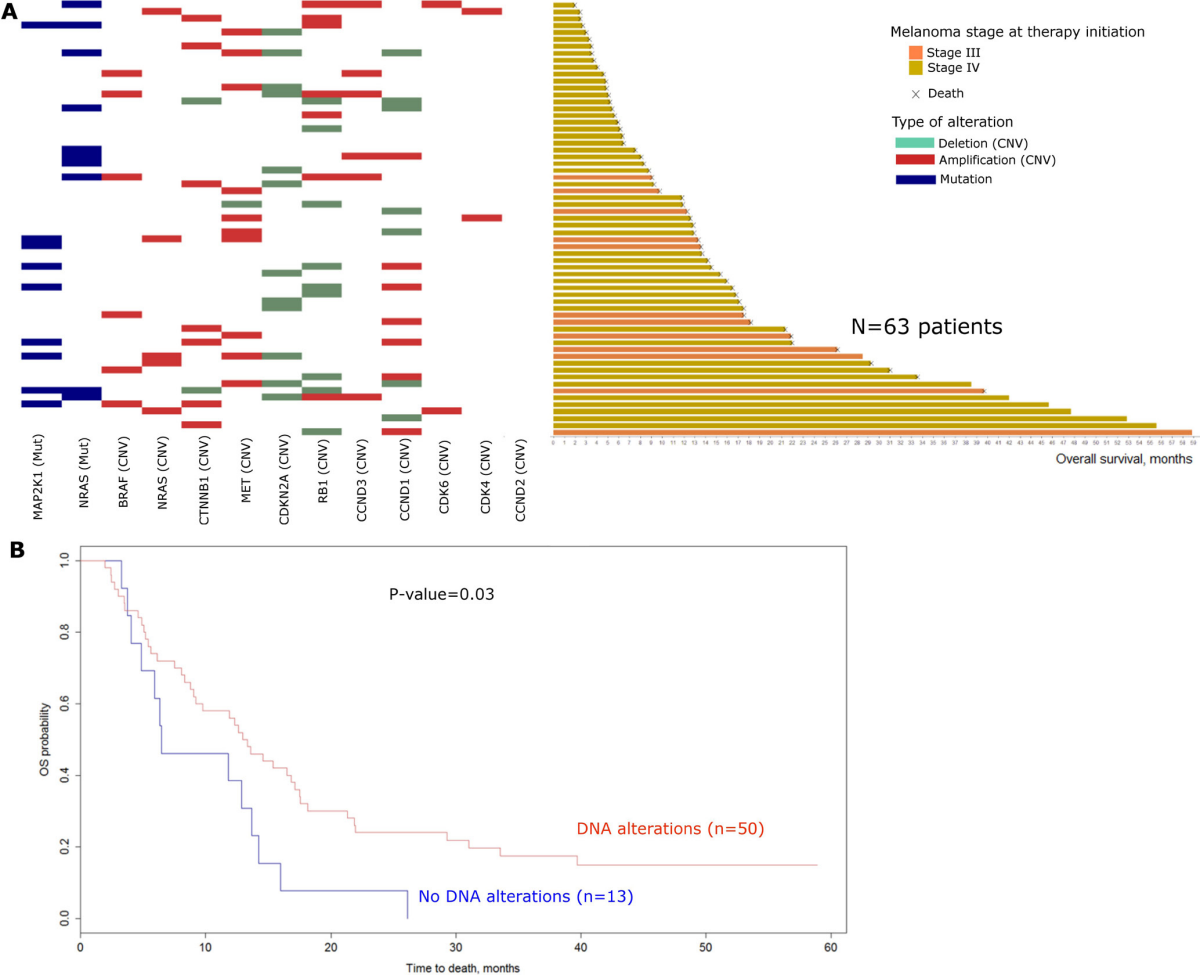
Baseline DNA alterations: (**A**) Landscape of baseline DNA alterations for the 63 patients with available data (1 missing). Patients are ranked according to their overall survival. Mutations are represented in blue, Amplifications and deletions are red and green respectively. (mut: mutations; CNV: Copy number variations). (**B**) Kaplan Meier curves for overall survival comparing patients with at least one DNA alteration at baseline (*n* = 50) versus patients with no DNA alteration at baseline (*n* = 13). Log-rank test was performed to compute the *P*-value (Univariate analysis). (OS: Overall survival).

DNA analysis (copy number variations and mutation analysis) of the 12 studied genes (*BRAF, NRAS, MAP2K1, MET, CDKN2A, CDK4, CDK6, CCND1, CCND2, CCND3, RB1, CTNNB1*) (Supplementary Table 2) was performed in melanoma lesions from 63 patients before treatment (1 missing because of insufficient material). This targeted analysis allowed the detection of 102 alterations, including 83 copy number variations (CNVs) and 19 mutations (Supplementary Table 3). *NRAS* mutations were identified in 10 samples (15.9%) and *MAP2K1* mutations in 9 samples (14.3%) including concomitant mutations of *NRAS* and *MAP2K1* in 2 samples. Copy number variations on *RB1* were the most frequent DNA alterations observed with deletions and amplifications in 9 (14.3%) and 7 (11.1%) out of 63 samples respectively. Among the 63 patients evaluated, 35 (55.6%) were defined as responders (partial or complete response) to BRAF inhibitors and 28 (44.4%) as non-responders (stable or progressive disease). According to the DNA analysis on the 12 screened genes, the number of alterations was not significantly different in these two groups with an average of 1.51 and 1.75 alterations (CNVs or mutations) per sample in responders and non-responders respectively. Similarly, number of alterations was not significantly associated with PFS or OS. Figure 2A presents DNA alterations detected in the 12 studied genes for the 63 patients ranked according to their OS. A binary variable was constructed (at least one detected DNA alteration *vs* no detected alteration in our studied genes) and univariate survival analysis was performed. Overall survival was found significantly higher (*p* = 0.03, Figure 2B) in patients with at least one DNA alteration (CNVs or mutations, *n* = 50) *vs* patients with no DNA alteration (*n* = 13). Despite the absence of significance, a similar trend was described for PFS. Sex and presence of brain metastasis were selected among the clinical baseline candidate variables for further adjustments. The trend observed in univariate analysis was maintained in multivariate analysis but no significant association between the detection of at least one DNA alteration and PFS/OS was observed.

Despite a lack of significance after adjustments, these data suggest that the presence of at least one underlying DNA alteration among our studied genes at baseline may be in favor of a better clinical course under BRAF inhibitors.

### mRNA overexpression of genes’ subsets prior to treatment is associated with improved PFS and OS in melanoma patients under BRAF inhibitors

**Figure 3:**
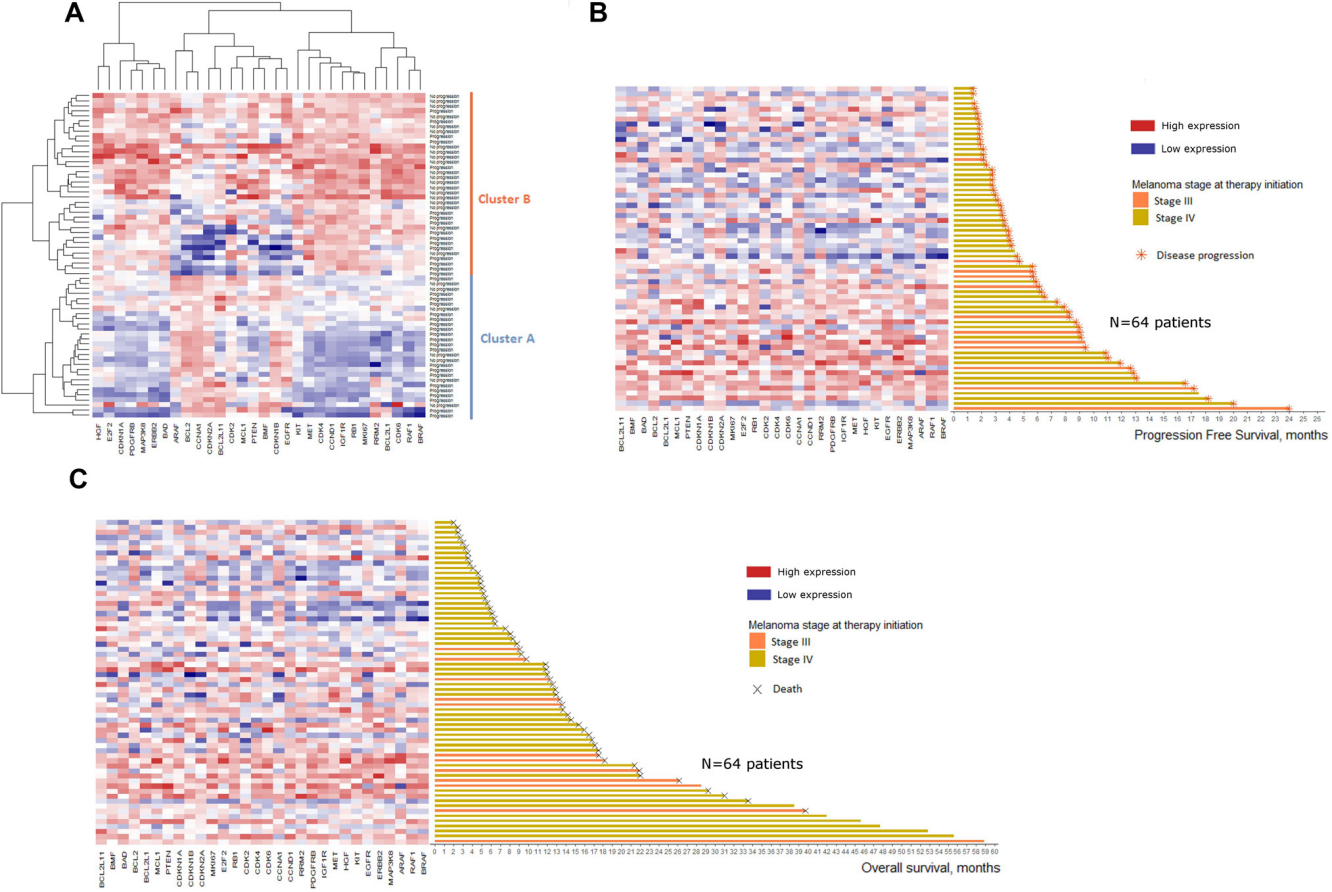
Heatmaps of baseline mRNA expression for the 64 patients included Color represents the relative expression of each gene in each sample, centered on the mean and scaled to the standard deviation. Blue is low expression and red is high expression. (**A**) Heatmap of unsupervised clustering. Patients are defined according to their progression status 6 months after therapy initiation (Progression *vs* No progression). (**B**) Heatmap of supervised clustering. Patients are ranked according to their progression free survival. (**C**) Heatmap of supervised clustering. Patients are ranked according to their overall survival.

mRNA analysis was performed on 30 genes involved in RAS-RAF-MAPK pathway, cell cycle or apoptosis and were implicated in BRAF inhibitors resistance mechanisms (Set 1: *BRAF, RAF1, ARAF, PDGFRB, IGF1R, MET, HGF, KIT, EGFR, ERBB2, MAP3K8, MKI67, E2F2, RB1, CDK2, CDK4, CDK6, CCNA1, CCND1, RRM2, BCL2, BCL2L1, BCL2L11, BMF, MCL1, BAD, PTEN, CDKN1A, CDKN1B, CDKN2A*) (Supplementary Table 2). Gene expression data are presented in Supplementary Table 3. An unsupervised analysis was conducted to identify mRNA expression profile susceptible to predict the clinical course under BRAF inhibitors. A heatmap was generated and differentiated two clusters with distinct mRNA profiles (Figure 3A). Patients in Cluster A who had lower mRNA expression levels of the studied genes were more likely to develop disease progression at 6 months (21/28, 75%) than patients in cluster B with higher mRNA expression levels (16/36, 44%). The distribution of patients with at least one DNA alteration was not significantly different in the two clusters (Fisher's exact test, *p* = 0.22).

Ranking the 64 patients according to their PFS or OS confirmed this trend with an increased mRNA expression for patients with higher PFS (Figure 3B) and higher OS (Figure 3C).

Cox scores for each gene and for both time-to-event endpoints were obtained from supervised principal components analysis and showed protective association with survival (Supplementary Figure 1). Using the genes with the highest absolute Cox scores (*i.e.* highest correlation with survival), 2 gene signatures were computed: 1) PFS gene signature composed of 11 genes highly correlated with PFS (Set 2: *ERBB2, CDKN1A, BAD, BRAF, EGFR, BMF, MAP3K8, E2F2, RAF1, CDKN1B, CDK6*) and 2) OS gene signature composed of 10 genes highly correlated with OS (Set 3: *CCND1, CDK4, IGF1R, MKI67, CDKN1A, PDGFRB, ERBB2, MAP3K8, BAD, CDK6*). Two mRNA expression profiles with high prognostic potential for PFS and OS were constituted with these two gene signatures and led to two Cox multivariate models including sex and presence of brain metastasis as clinical variables.

**Figure 4:**
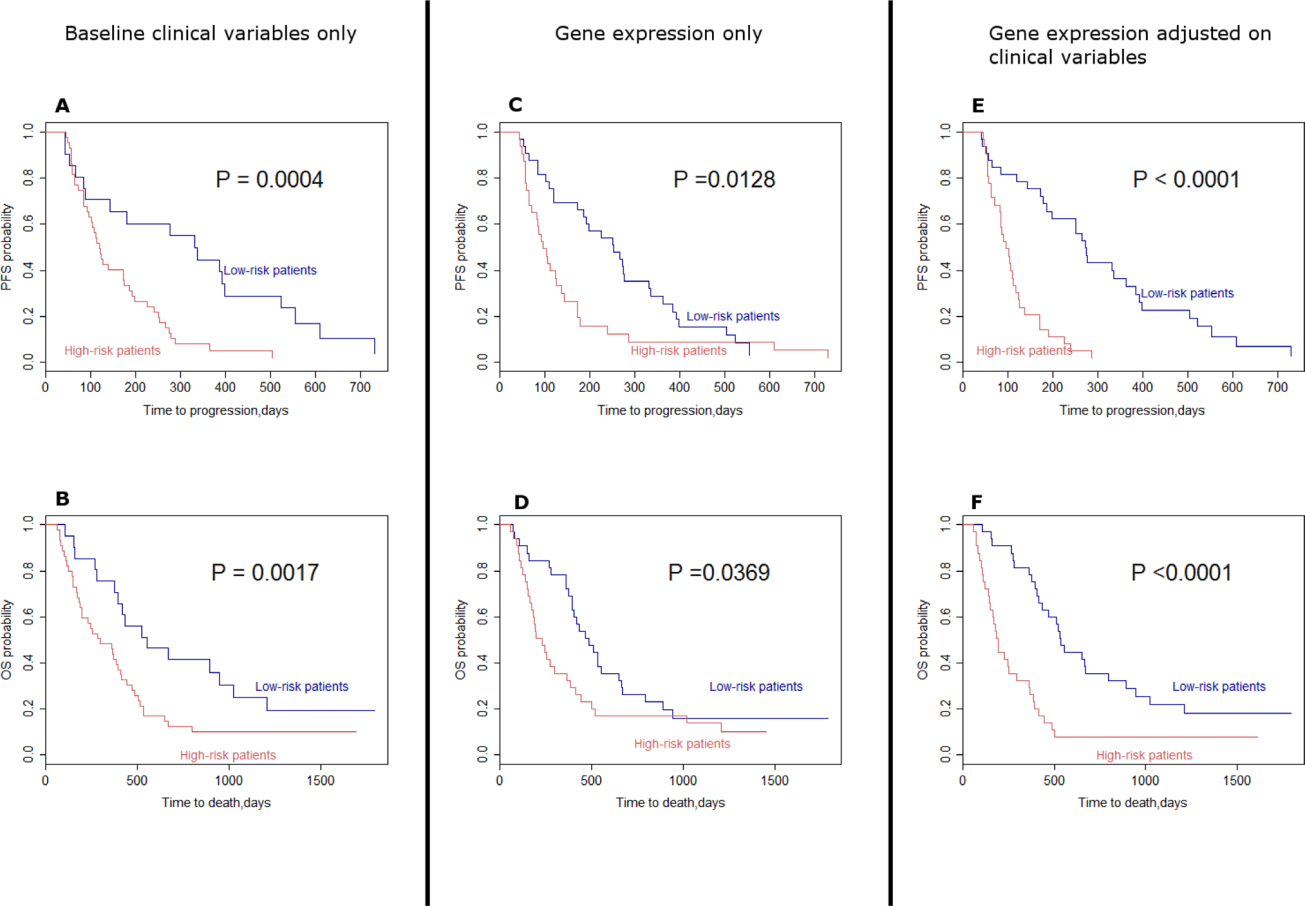
Kaplan–Meier curves assuming the survival in high risk patients and low-risk patients (**A**) Progression free survival associated with selected baseline clinical variables (sex and presence of brain metastasis). (**B**) Overall survival associated with selected baseline clinical variables (sex and presence of brain metastasis). (**C**) Progression free survival associated with gene expression only. (**D**) Overall survival associated with gene expression only. (**E**) Progression free survival associated with gene expression adjusted on selected clinical variables. (**F**) Overall survival associated with gene expression adjusted on selected clinical variables. Log-rank tests were performed to compute *P*-values. (OS: Overall survival; PFS: Progression free survival).

The cohort was divided into high and low risk subgroups based on prediction using 1) the baseline clinical variables only (sex and presence of brain metastasis), 2) the gene expression profile only and 3) the gene expression profile adjusted on sex and presence of brain metastasis. The model with only the baseline clinical variables was a good discriminator for PFS (*p* = 0.0004, Figure 4A) and OS (*p* = 0.0017, Figure 4B). Similarly, the gene expression signature alone was a significant discriminator of PFS (*p* = 0.0128, Figure 4C) and OS (*p* = 0.0369, Figure 4D). As expected, considering both gene expression and clinical variables considerably increased the prognostic power, highlighting the interest of taking into account these two types of variables to predict PFS (*p* < 0.0001, Figure 4E) and OS (*p* < 0.0001, Figure 4F).

To provide further arguments regarding the external validity of our findings, analyses were conducted on a baseline gene expression dataset of metastatic melanoma patients treated with BRAF inhibitors as monotherapy, in a similar setup of our retrospective study. The patient dataset GSE50509 from Rizos *et al.* [15] publicly available on GEO Datasets, which described 21 metastatic melanoma patients treated with either vemurafenib or dabrafenib as monotherapy, was selected. This dataset by Rizos *et al.* included baseline gene expression data for 34 078 *loci* obtained using Illumina Human HT-12 V4.0 expression beadchip and clinical variables. Using this set, we first computed a heatmap focusing on the 30 genes of our panel and patients were ranked according to their PFS. A similar trend to that obtained with our patients’ series was observed with a selection of genes showing increased mRNA expression for patients with higher PFS (Supplementary Figure 2). Despite a moderate significance, gene set enrichment analysis (GSEA) performed on set 2 (PFS gene signature) and set 3 (OS gene signature) on Rizos *et al.* dataset showed enrichment and upregulation of mRNA expression for favorable phenotypes in set 2 (*p* = 0.019 and *p* = 0.096 for categorical and continuous labeling respectively) and in set 3 (*p* = 0.071 and *p* = 0.037 for categorical and continuous labeling respectively). Enrichment plots of GSEA are presented in Supplementary Figure 3.

### Genomic alterations at relapse uncovers a large landscape of resistance mechanisms to BRAF inhibitors

Tumor samples at relapse were available for 20 patients. Among these 20 patients, 5 (25%) had collection of multiple samples. In total, 30 relapse related biopsies were analyzed to set a landscape of acquired resistance mechanisms. This series of 20 patients was comparable to the rest of the cohort in terms of clinical and pathological characteristics and was homogenously distributed in the previously described clusters, suggesting an absence of selection bias. As observed in our cohort, OS and PFS in this subgroup were improved in patients with the higher mRNA expression profile and presenting at least one DNA alteration at baseline.

**Figure 5:**
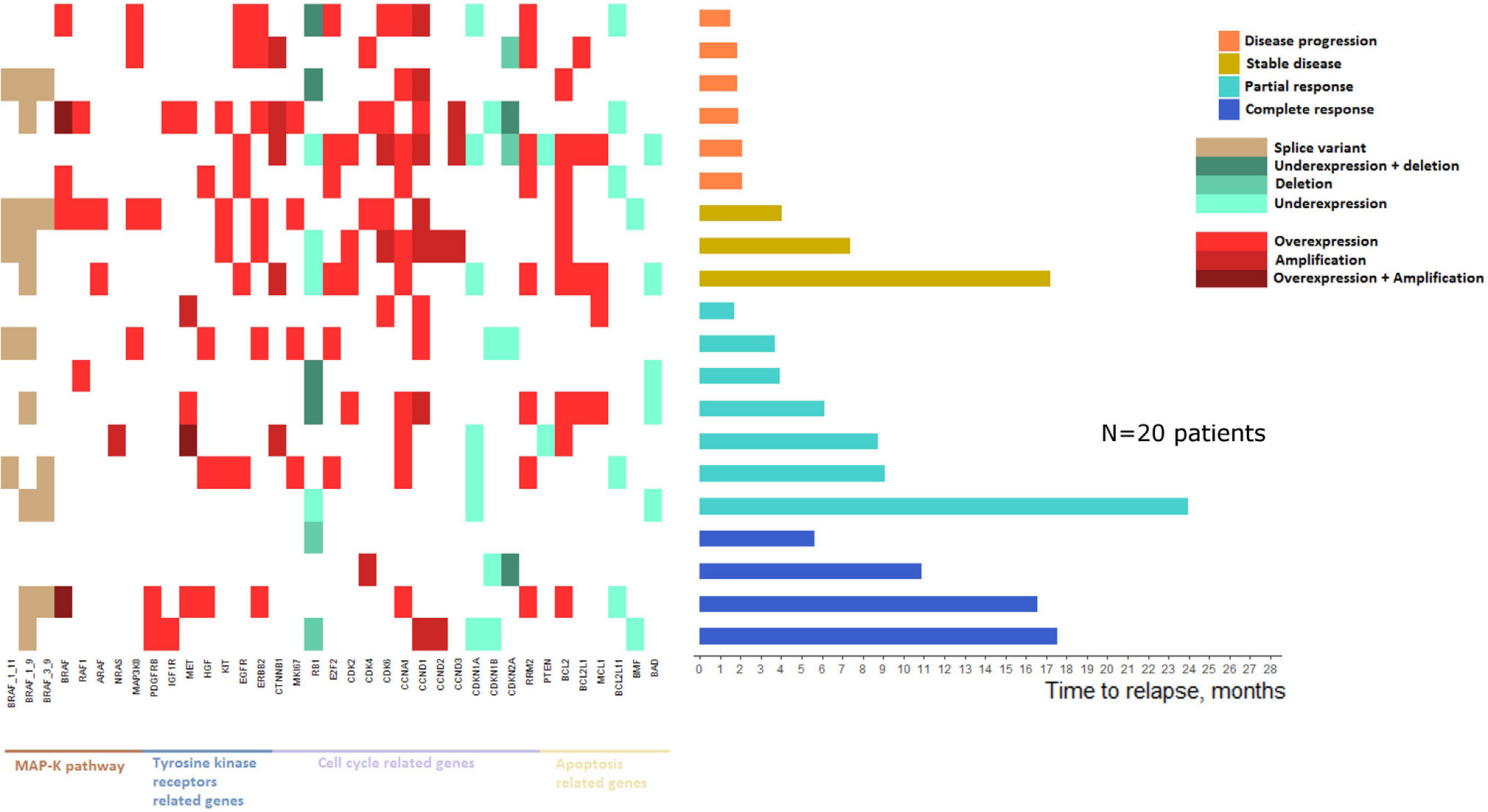
Landscape of mRNA expressions and DNA alterations at relapse reported to baseline status

Changes of CNVs and mRNA expression at relapse reported to baseline status are presented in Figure 5. Patients were ranked according to their best observed response and their time to relapse. For patients with multiple collected lesions, the earliest post-relapse sample was used to compute the landscape of mRNA and DNA alterations.

Patients with a worse clinical course were more likely to present mRNA and DNA alterations and at least one resistance mechanism was identified in tumors from all relapsed patients. Thus, mRNA/DNA alterations at relapse were significantly higher in patients with no clinical response (*i.e.* patients with disease progression or stable disease as best observed response) than patients presenting a clinical response (*i.e.* patients with partial or complete response as best observed response with a mean of respectively 12.2 and 6.8 alterations (Wilcoxon test, *p* = 0.01).

Sixteen patients presented a resistance mechanism implicating alterations in MAPK pathway related genes. Among them, *BRAF* splice variants (1-11, 1-9 and 3-9) were identified in 11 patients. Moreover, among the 4 patients not showing alterations in the MAPK pathway related genes, 3 presented a clinical response. Similarly, 15 patients presented alterations in tyrosine kinase receptors related genes and *ERBB2* amplification was the most frequent alteration (*n* = 7). Four of the 5 patients with no tyrosine kinase receptors related genes alterations presented a clinical response. Apoptosis related genes were also altered in 17 patients with *BCL2* amplification as the most frequent alteration (*n* = 9). The 3 patients with no alterations in this subset presented a partial or complete response. Only two patients showed no alterations in any of MAPK pathway, tyrosine kinase receptors and apoptosis related genes, and interestingly, these two patients both presented a complete response lasting 5 and 11 months.

### Genomic alterations at relapse reveal *RB1* downregulation as a potential mechanism of resistance to BRAF inhibitors

**Figure 6:**
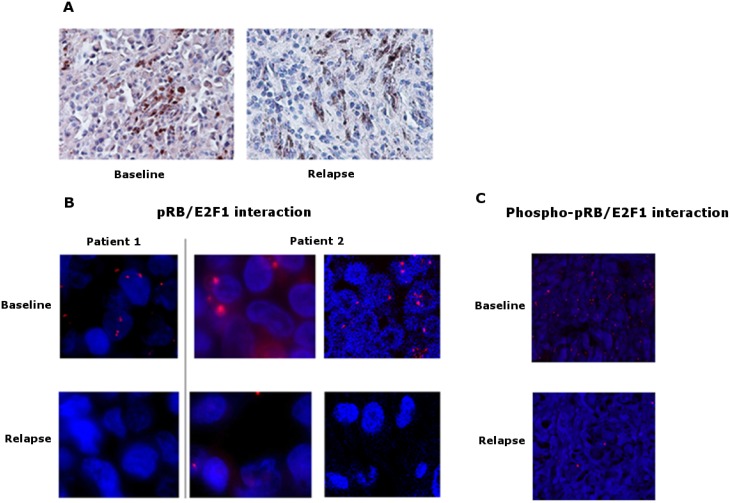
pRB expression and pRB/E2F1 interaction in tumors (**A**) Representative pictures of immunohistochemical staining of pRB on sections of baseline and relapse tumor samples. Baseline sample shows a slight diffuse cytoplasmic staining versus no staining in the relapse sample, magnification ×400. (**B**) Representative pictures of *in situ* proximity ligation assay (PLA) demonstrating pRB and E2F1 interaction in tumor sections at baseline and relapse from 2 melanoma patients. pRB-E2F1 heterodimerization was visualized as red dots by *in situ* PLA and was detected with a fluorescent Axiovert microscopy # patient 1 and fluorescent Axiovert (left) and confocal microscopy (right) # patient 2; cell *nuclei* were stained with DAPI (blue), magnification ×63. (**C**) Representative pictures of *in situ* proximity ligation assay (PLA) demonstrating phospho-pRB and E2F1 interaction in tumor sections at baseline and relapse from a melanoma patient. Phospho-pRB-E2F1 heterodimerization was visualized as red dots by *in situ* PLA and was detected with confocal microscopy; cell *nuclei* were stained with DAPI (blue), magnification ×20.

**Figure 7:**
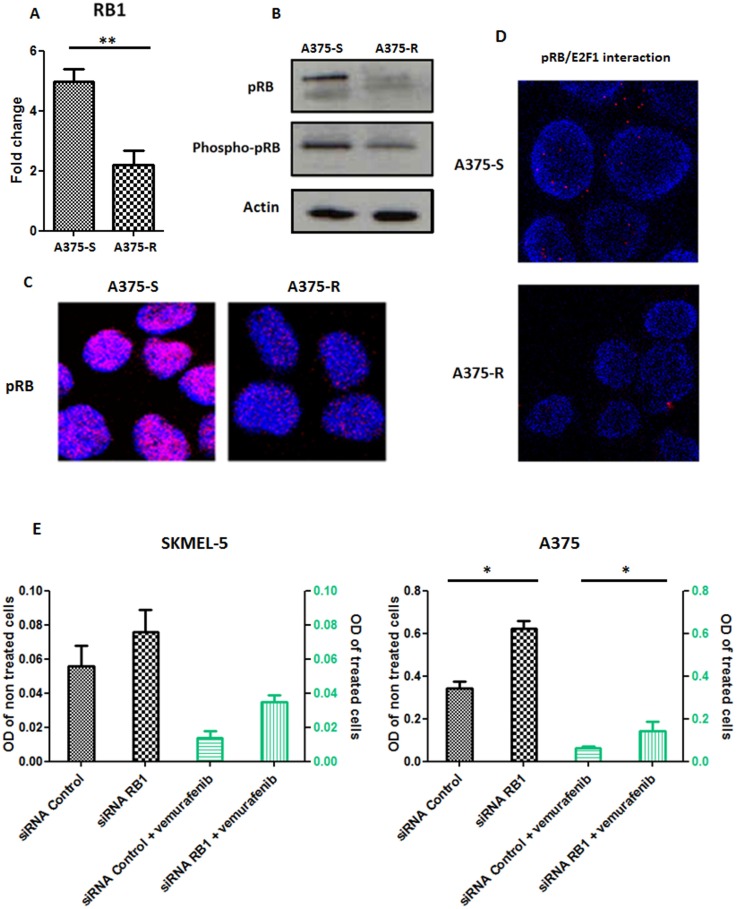
*In vitro* studies of RB1 (**A**) RB1 transcript expression in vemurafenib sensitive A375 cells (A375-S) and resistant A375 cells (A375-R). ^**^p < 0.001. Bars represent means from three independent experiments. (**B**) Western blot analysis of the levels of phospho-pRB, pRB in the vemurafenib sensitive parental A375 cells and vemurafenib resistant A375 cells. Actin was used as internal control. Representative blots of three independent experiments are shown. (**C**) Representative pictures of expression of pRB in vemurafenib sensitive and resistant A375 cells assessed with confocal microscopy; cell *nuclei* were stained with DAPI (blue); magnification ×63. (**D**) Representative pictures of *in situ* proximity ligation assay (PLA) demonstrating pRB/E2F1 interactions in A375 melanoma cells sensitive (A375-S) or resistant (A375-R) to vemurafenib. pRB-E2F1 heterodimerization was visualized as red dots by *in situ* PLA and was detected with a confocal microscopy; cell *nuclei* were stained with DAPI (blue); magnification ×63. (**E**) Proliferation assay in SKMEL-5 and A375 cell lines undergoing a RB1 downregulation with RNA interference and treated or not with vemurafenib 1 μM. Bars represent means from three independent experiments. ^*^p < 0.01 (siRNA Control: small interfering RNA control; siRNA RB1: small interfering RNA RB1, OD: Optical density).

All patients underwent mRNA/DNA alterations in the cell cycle related genes, underlining the implication of this pathway in relapse whatever the best observed clinical response. Among these genes, *RB1* showed the most frequent rate of alterations (10/20, 50%). Interestingly, only *RB1* deletion was detected in one of the relapsed lesions in a patient who showed a complete response at 2 months but relapsed at 6 months. To explore the resistance phenotype conferred by *RB1* gene downregulation, RB1 protein (pRB) level and its interaction with E2F1 transcription factor were analyzed by immunohistochemistry and *in situ* proximal ligation assay (PLA) respectively, in 7 matched pairs of tumors at baseline and relapse. pRB expression and pRB/E2F1 interaction were significantly decreased in relapse melanoma lesions compared to baseline (Figure 6A and 6B). Furthermore, the interaction between pRB Ser-807-811 phosphorylated form (Phospho-pRB), which is not required to inhibit E2F1 binding [21], and E2F1 was similarly decreased by PLA analysis, confirming the alteration in pRB/E2F1 interaction at relapse (Figure 6C). *In vitro* studies using a vemurafenib-resistant melanoma A375 cell line have shown a lower expression of *RB1* transcripts and pRB than the vemurafenib-sensitive A375 cells (Figure 7A–7C and Supplementary Figure 4A). A decrease of pRB/E2F1 interaction was also observed in the resistant A375 cells compared with the sensitive cells (Figure 7D). *RB1* downregulation by RNA interference led to vemurafenib resistance in A375 and SKMEL-5 cell lines, increasing proliferation rate (Figure 7E) and no significant variation in proliferation rate was observed with cyclosporin (immunosuppressant reported to inhibit melanoma cell proliferation [22]), suggesting the implication of RB1 in mediating vemurafenib resistance (Supplementary Figure 5). Moreover, *RB1* mRNA expression was assessed in two melanoma cell lines (SKMEL-5 and COLO829) after 3 days of vemurafenib treatment and the most sensitive SKMEL-5 cells presented the highest *RB1* expression compared to COLO829 cells (Supplementary Figure 6).

This targeted approach combining profiles of mRNA and DNA alterations involved in MAPK inhibitors mechanism of action, allowed the identification of resistance mechanisms for all metastatic melanoma patients tested.

### Intra-patient heterogeneity of gene alterations

**Figure 8:**
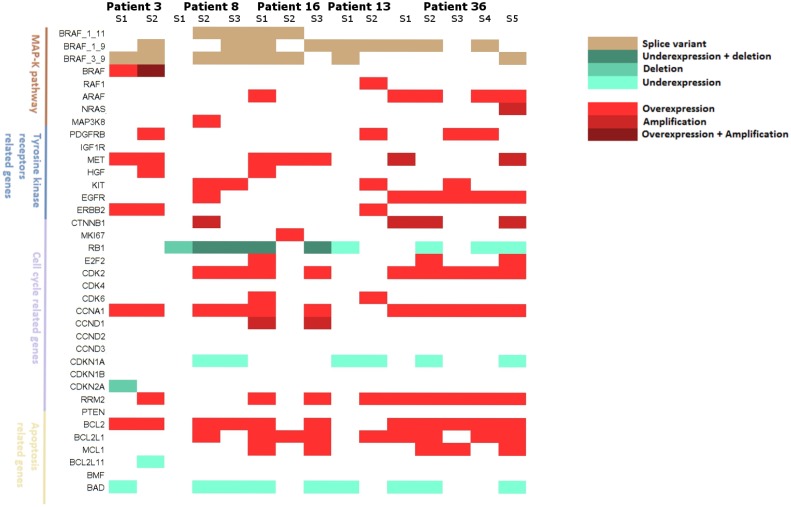
Landscape of resistance mechanisms addressed by patients with collection of multiple relapse samples Patients 3 and 13 had 2 relapsed biopsies, Patients 8 and 16 had 3 relapsed biopsies and patient 36 had 5 relapsed biopsies. mRNA expressions and DNA alterations at relapse are reported to baseline status.

Five patients had multiple relapse samples allowing the assessment of tumor heterogeneity. Two patients had 2 biopsies, 2 had 3 biopsies and 1 patient had 5 biopsies (Figure 8). Multiple alterations co-occurred in 14 of 15 tumor samples (93.3%), highlighting the probable complementary role of these alterations. At the signaling pathway level, the four studied pathways (MAPK pathway, tyrosine kinase signaling, cell cycle and apoptosis) were quasi-systematically altered (13 out of 15 samples, 86.7%), while the cell cycle pathway was altered in all tumor samples. Patient 3 and patient 16 harbored wide gene alteration heterogeneity and all pathways were affected in each tumor sample. Patient 13 and 36 presented the same trend with 3 of 4 pathways systematically altered in distinct tumor biopsies. Interestingly, patient 8 had 1 tumor sample harboring only cell cycle deregulation through *RB1* deletion confirming its major role in resistance to BRAF inhibitors.

## DISCUSSION

In this study, we developed a targeted genomic approach combining DNA and mRNA expression analysis which allowed the identification of acquired resistance mechanisms to BRAF inhibitors in all available relapse samples. Previous studies, which mainly focused on exome analysis, associated or not to mRNA analysis, have shown that over 25% relapsed BRAF inhibitor patients could not be attributed any known resistance mechanism. Therefore, our genomic analysis emerges as an interesting approach to identify a larger spectrum of resistance mechanisms. Among the observed mechanisms detected in our study, 80% were involved in MAPK pathway reactivation at relapse, which is much higher than the 44% previously reported by Van allen *et al.* [19]. Moreover, with our mRNA expression approach, we were able to identify combined resistance mechanisms in 19/20 (95%) patients involving several pathways and to bring new insight regarding acquired resistance to BRAF inhibitors. It is important to underline that, since resistance mechanisms to the combination of BRAF and MEK inhibitors appear to be very similar to those observed for BRAF inhibitors alone, [23, 24] our results could be relevant to such association therapy. Importantly, our study also underscores the value of *RB1* downregulation as an important and frequent alteration implicated in acquired resistance to BRAF inhibitor. Concurrent inactivation of *PTEN* and *RB1* have previously been described as a mechanism for loss of BRAF/MEK dependence in *BRAF^V600E^* mutated melanomas [25] and the cell cycle progression pathway including *RB1* have been associated to BRAF inhibitors resistance [26–28].

Another important issue highlighted in our study is the intra-tumor heterogeneity, which is further enhanced by clonal variation and remains a challenge in therapeutic management. Van allen *et al.* [19] have suggested the possible existence of subclonal population and it seems obvious now that a simple biopsy does not describe exhaustively the mutational load [15]. Johnson *et al*. [20], studying genetic mutations in several biopsies from the same patients, reported a genetic heterogeneity within individual patients with 10/19 patients harboring non-overlapping resistant mechanisms. The approach described here of combining resistance factors of different pathways at the mRNA and DNA levels has shown that while there was heterogeneity in the individual genetic alterations, these overlapped when analyzing them within their signaling pathways.

Regarding gene alterations prior to BRAF inhibitor initiation, the rate of *BRAF/NRAS* co-occurrent mutations observed in our cohort at baseline (15.9%) was comparable with the 11% described by Larkin *et al.* [29] and is explained by the high sensitivity threshold of our technique (0.5%). Moreover, the observation of baseline *NRAS* and *MAP2K1* mutations concomitantly with *BRAF* mutation in 17 (26.6%) patients are consistent with the 24% rate reported by Johnson *et al.* [20] in relapsed tumor samples and confirm the implication of these mutations in BRAF inhibitor resistance. Although previous studies have already focused on the measurement of mRNA expression on relapse tumors [15, 19, 30, 31], only few addressed baseline levels prior to treatment with a MAPK inhibitor.

Our targeted approach analyzing both DNA alterations and mRNA expression reveals the relevance of a subset of pre-existing genetic alterations as a predictive marker of improved clinical benefit achieved under BRAF inhibitor therapy.

We show that the more the tumors harbor genetic alterations in the screened genes the better the response to BRAF inhibitors would be. Indeed, despite a lack of significance, the presence at baseline of at least one DNA alteration in our screened genes was in favor of a better clinical course. In addition, tumor mRNA analysis prior to treatment have shown 2 sets of genes with higher levels of transcript expression which were significantly associated with improved PFS and OS. Despite the moderate significance of our findings which can be explained by the size of our cohort, the same trend was observed from the GSEA analysis performed on the dataset from Rizos *et al.* [15] and provide an external validation of our gene signatures confirming its relevance as baseline predictors of clinical response in patients treated with BRAF inhibitors.

Interestingly, both oncogenes and tumor suppressor genes are upregulated in our signatures which may reflect different proliferation/metabolic rate of the tumor, rendering it more sensitive to treatment. This underlines the complexity of the mechanism of action of BRAF inhibitors. In this context, a study focusing on tumor samples prior to BRAF inhibitor initiation in metastatic melanoma patients showed improved survival in patients with an immune gene overexpression signature which included both activating and suppressive immune regulators [32]. Furthermore, using co-BRIM study (vemurafenib arm) data set, Wagle *et al.* [33] also proposed a MAPK Pathway Activity score (MPAS) based on the expression of 10 genes involved in MAPK pathway activity (including suppressor genes and oncogenes) as a relevant predictor of clinical response to vemurafenib, and a high score (i.e. higher MAPK pathway signaling) was associated with improved progression free survival, which is consistent with our results.

These observations are also consistent with recent studies on metastatic melanoma patients undergoing immunotherapy where, using WES, a high mutational load was associated with improved clinical course [34, 35]. Regarding BRAF inhibitors specifically, Trunzer *et al.* [36] have shown a significant association between high expression of *PTEN* and response to vemurafenib in metastatic melanoma patients. Rizos *et al.* [15] have noticed a significantly better PFS in patients with reactivation of the MAPK pathway compared to patients with a persistent MAPK inhibition at relapse, highlighting the complex activity of BRAF inhibitors and arguing that it may reflect the partial cytostatic activity of this therapy [37]. More recently, a higher overall mutation rate at baseline has been associated with longer OS in melanoma patients treated with dabrafenib + trametinib [38]. Hence, the multiparametric profile of mRNA and DNA alterations involved in the mechanisms of action of MAPK inhibitors supports the benefit of pre-existing genetic and genomic alterations in the improvement of clinical response to BRAF targeted therapies.

Despite the retrospective nature of our study, our cohort provided data representative of the clinical practice and the clinical and pathological characteristics were consistent with clinical trials [1–3, 39]. Studied genes included in our present targeted approach have been chosen by screening the literature and selected according to their potential influence in the resistance to BRAF inhibitor therapy, including tyrosine kinase receptor related genes, MAPK pathway genes, cell cycle genes and apoptosis related genes [37, 40]. Our approach appears as a relevant tool to be implemented in clinical practice as it can be adapted to new targeted therapies by including newly identified genetic alterations.

As the huge promise of immunotherapy in melanoma may lead in the near future to combination or sequential treatment with MAPK targeted agents and as molecular mechanisms of resistance to immunotherapy are also emerging, future strategies will require the comprehension of the combined resistance factors for both targeted therapies and immunotherapy in order to improve the clinical management of patients. In this respect, the approach employed here which enlarges the identification of resistance mechanisms to BRAF inhibitors can be extended to genes involved in immunity. As highlighted by Hugo *et al.* [12], taking into account genes involved in intra-tumoral immunity would be of great interest to further explore MAPK inhibitor resistance. Using microarray gene expression analysis which included immune genes, Lardone *et al*. [30], Mann *et al.* [31] and Mandruzzato *et al.* [41] identified gene signatures predictive of PFS or OS in metastatic melanoma patients and found that the expression of immune related genes was associated with improved clinical outcome. Nevertheless, these studies did not focus on BRAF inhibitors treated patients. More recently, a study conducted by Wongchenko *et al.* [32] in metastatic melanoma patients treated with BRAF inhibitors, has associated a higher baseline expression of immune regulatory genes to an improved PFS. However, genes evaluated in this study were not specifically selected according to their relevance regarding resistance mechanism to BRAF inhibitors.

Our approach combining DNA and mRNA alterations analysis emerges as a relevant tool to identify those patients who will achieve durable response to targeted therapies in clinical practice and to provide an exhaustive description of acquired resistance mechanisms to these therapies to a better management of such resistance.

## MATERIALS AND METHODS

### Patients and tumor samples

From 2010 to 2013, 64 patients (59 from the onco-dermatology department of Saint Louis hospital, Paris, France and 5 from the onco-dermatology department of the Lyon Sud hospital, Lyon, France) with a stage III or IV metastatic melanoma were included in this retrospective study. All patients presented *BRAF^V600^* mutated lesions at inclusion and were treated with either vemurafenib or dabrafenib as a monotherapy. Baseline was defined as the initiation time of targeted therapy. Patients were under care of a dermato-oncologist in order to assess response, progression or relapse and to detect resistance to BRAF inhibitors as described by Fennira *et al.* [42]. Type and date of best response under targeted therapy were obtained from patient's medical records and evaluated using RECIST (Response evaluation criteria in solid tumors) [43]. Tumor samples were collected at baseline for each patient and at relapse in a subset of patients undergoing a biopsy as part of routine care and stored as formalin-fixed paraffin embedded (FFPE) or frozen. Samples harboring below to 50% of tumor cells were macrodissected.

### DNA/mRNA extraction and reverse transcription

DNA and mRNA extraction were performed on tumor samples. DNA extraction was performed with QIAamp DNA FFPE Tissue kit (Qiagen, Hilden, Germany) according to the manufacturer's protocol. Total RNA was isolated using Trizol reagent (Thermo Fisher Scientific, Waltham, USA) and RNeasy FFPE kit (Qiagen, Hilden, Germany) according to the manufacturer's protocol. DNA and mRNA were qualified and quantified using a NanoDrop ND-1000 spectrophotometer (NanoDrop Technologies, Wilmington, USA). DNA quantification was also performed with a Qubit 2.0 fluorometer (Thermo Fisher Scientific, Waltham, USA). First-strand cDNA was synthetized with a high-capacity cDNA reverse transcription kit (Life technologies, USA).

### Mutation analysis on *BRAF*, *NRAS*, *MAP2K1* genes

*BRAF* V600 genotype was characterized by the COBAS 4800 *BRAF^V600^* Mutation Test on a LightCycler 480 (Roche, France) and by pyrosequencing using a PyroMark-96 MD pyrosequencer (Qiagen, Hilden, Germany) as previously described (sensitivity of 5% for both methods) [44, 45]. *NRAS* G12, G13 and Q61 genotype were characterized by High Resolution Melting curve (HRM) analysis with a sensitivity of 5% [46] and E-ice-cold-PCR on a LightCycler 480 (Roche, France) followed by pyrosequencing on a PyroMark-96 MD pyrosequencer (Qiagen, Hilden, Germany) with a sensitivity of 0.5%, as previously described [45, 47]. *MAP2K1* exons 2 and 3 mutation analysis was performed with a BigDye Terminator v1.1 sequencing kit on a 3130XL analyzer (Applied Biosystems, USA) [48].

### Copy number and mRNA expression analysis

Gene copy number variation (CNV) and mRNA expression analysis was performed by quantitative PCR (qPCR) using the off-the-shelf commercial personalized Human qPCR SignArrays^®^ 96 system (qPCR SignArrays^®^ 96 VPR1H1 kit, Anygenes, France). A total volume of 20 μl PCR mix, including 10 μl of Perfect MasterMix SYBR Green^®^, 8 μl of PCR grade water and 2 μl of DNA (or complementary DNA after reverse transcription) was dropped into each well of the qPCR array and analyzed with LightCycler 480 (Roche, France). PCR amplification was conducted in duplicates at 95° C for 10 minutes, followed by 40 cycles of 95° C for 10 seconds and 60° C for 30 seconds. Studied genes are involved in RAS-RAF-MAPK pathway, cell cycle or apoptosis and were selected for their validated or suggested role in BRAF inhibitors resistance (Supplementary Table 2) [40]. Overall, mRNA expression analysis was performed on 30 genes and copy number analysis on 11 genes.

mRNA expression analysis was performed after reverse transcription and normalization using the average expression of one relevant housekeeping gene. Six genes were candidate for normalization: *peptidylprolyl isomerase A (cyclophilin A, PPIA), b-actin (ACTB), TATA box binding protein (TBP), beta- 2-microglobulin (B2M), hypoxanthine phosphoribosyltransferase 1 (HPRT1) and transferring receptor (p90, CD71) (TFRC).* Finally, *peptidylprolyl isomerase A (cyclophilin A, PPIA)* was selected as the most relevant gene due to its stability in our tissue-set. mRNA expression for each gene was expressed as the ratio (copy number of gene of interest/copy number of *PPIA*).

Gene copy number quantification was performed by comparison with *Glyceraldehyde 3-Phosphate Deshydrogenase* (*GAPDH*), using 2 sets of primers for each gene, as described previously [49]. Relative copy numbers were calculated using the ΔΔCt method, where Ct is the threshold cycle of amplification. For each sample, differences in the Ct of targeted gene and *GAPDH* used as an internal control were compared with those in a reference pool of normal genomic DNA prepared from 10 samples of benign tissue. Relative copy number was calculated using the formula 2^(−ΔΔCt)^ and converted to absolute copy numbers by assigning a value of 2 (diploid) to the reference pool and multiplying the relative copy number of samples by a factor of 2. Threshold of 5 and 0.5 were set to define DNA amplification and DNA deletion respectively [50, 51]. Change of CNVs and mRNA expression at relapse were expressed as fold change between relapse and baseline specimens. A change > 2 or < −2 was considered respectively as a significant increase or decrease of CNVs or mRNA expression.

### Cell culture, reagents and siRNA transfection

Human melanoma A375 (primary melanoma) and SKMEL-5 (metastasis lymph node melanoma) BRAF V600E, c.1799T>A mutated cells were acquired from the American Type Culture Collection (ATCC, (Manassas, VA)). Cells were cultured in DMEM supplemented with 10% Fetal Bovine Serum (FBS), 100 U/ml penicillin and 100 mg/ml streptomycin, at 37° C in a humidified incubator with 5% CO2. Prior to functional studies, genotyping analysis showed that A375 bears wild-type p53, *CDK4* and *CDKN2A* 1α gene status; harbors a *CDKN2A* exon1β (p16) homozygous deletion and a *CDKN2A* exon2 E61X deleterious mutation, confirming functional status of *CDK4* able to phosphorylate RB1 protein.

Vemurafenib (PLX4032) was purchased from Selleckchem (Houston, Texas). Vemurafenib-resistant cells were chronically obtained by culturing A375 cells in increasing concentrations of vemurafenib for at least three months, as previously reported [52]. The selected resistant cells (A375-R) increased vemurafenib IC50 compared to the parental cells. In addition, under vemurafenib treatment, a strong activation of phospho-ERK and Cyclin D1 (CCND1) level was noted in the resistant A375-R cells, comparing to the parental A375 cells (Supplementary Figures 4B and 7). A375-R cells were further propagated in growth medium containing 2.5 μM of vemurafenib.

A375 cell transfections were carried out 24 hours after seeding cells on 6 well-plates (50%–60% confluent). RB1 siRNA (ON-TARGETplus SMART siRNA RB1, Dharmacon) or control siRNA (ON TARGETplus Non targeting, Dharmacon) were transfected into cells with Lipofectamine-2000 (Life Technologies) according to the manufacturer's protocol. Cells were then incubated for 24 h prior to treatment with vemurafenib and were then analyzed by Western Blotting, *In situ* proximity ligation assay, immunofluorescence and cell proliferation assay.

### Immunohistochemistry

Immunochemistry was performed on FFPE melanoma lesions at baseline and relapse. Tumor samples were successively deparaffinized in xylene, dehydrated through graduated alcohol series, treated with peroxidase 3% H_2_O_2_ block and incubated one hour at room temperature with mouse anti-pRB monoclonal antibody (Cell signaling, France) in Phosphate buffered saline (PBS) with 0.5% Bovine serum albumin (BSA). Negative controls were performed by replacing the primary antibody with PBS-BSA 0.5% alone. Samples were then incubated at room temperature for 30 minutes with biotinylated secondary antibody (Vectastain Elite universal ABC-kit (Vector-Laboratories, Burlingame)). Peroxidase activity was detected using 3-amino-9-ethylcarbazole at room temperature for 10 minutes and followed of counterstaining with Mayer's hematoxylin. Staining was visualized with a Nanozoomer 2.0-HT digital slide scanner (Hamamatsu, Japan) and images obtained with the NDPview2 software (Hamamatsu, Japan).

### Immunofluorescence, confocal microscopy

FFPE melanoma sections or vemurafenib sensitive and resistant A375 fixed cells, were incubated with primary anti-pRB antibody (Cell signaling, France) followed by Alexa Fluor 488 fluorescently conjugated secondary antibody. DAPI was used for nuclear counterstaining. Confocal and fluorescent images were taken with a laser-scanning confocal microscope (Leica Lasertechnik, Heidelberg) and using Axiovert fluorescent microscopy respectively.

### *In situ* proximity ligation assay

*In situ* proximity ligation assay (PLA) was performed to assess protein-protein interaction [53, 54]. Cells grown on 8-well culture slides (Labtek chamber slides (Nunc, #154534, Thermo Fisher) were fixed and subjected to *in situ* PLA using the Duolink Detection kit (Olink Bioscience, Sweden) according to the manufacturer's instructions. Briefly, melanoma tissue sections and fixed cell culture slides were incubated after blocking, with antibodies directed against pRB (Cell signaling, France), anti-phospho-pRb (Ser807-811) (Cell signaling, France) and E2F1 (Santa Cruz, France). Slides were thereafter incubated with PLA minus and PLA plus probes containing the secondary antibodies conjugated with oligonucleotides. Circularization and ligation of the oligonucleotides was followed by an amplification step. The products were detected by a complementary fluorescently labeled probe. Protein complexes were visualized using a laser-scanning confocal microscope (Leica-Lasertechnik) as bright fluorescent signals.

### Western blot

Whole-cell lysates were prepared in lysis buffer TBS-Nonidet P-40 1% containing 150 mM NaCl, 50 mM Tris buffer pH 7.5, 5 mM NaF, 0.2 mM Na3V04, phosphatase inhibitor (PhosStop, Roche) and complete protease inhibitor cocktail (Roche). Cell lysates were resolved by SDS-PAGE electrophoresis and transferred on nitrocellulose membranes. After incubation in blocking buffer, the membranes were probed with primary antibodies against pRB, phospho-pRb (Ser807-811), phospho-ERK1/2 (Thr202/Tyr204), ERK (Cell signaling, France) or actin (Abcam, France). The labeling was visualized using peroxidase-conjugated secondary antibodies and with an ECL kit (Pierce). All Western blots are a representative example of at least 3 independent experiments.

### Proliferation assay

Proliferation assays were performed on A375 and SKMEL-5 cell lines with or without vemurafenib (Selleckchem, PLX4032, S1267) or cyclosporin (Selleckchem, S1514) after siRNA transfection. Proliferation assay was also conducted on SKMEL-5 and COLO829 cell lines with or without vemurafenib (Selleckchem, PLX4032, S1267). Cell number was measured using the CellTiter 96 aqueous non-radioactive cell proliferation assay (Promega, France).

### Clinical baseline characteristics

Clinical characteristics included age at targeted therapy initiation, sex, melanoma subtype, Breslow thickness, melanoma stage, type of targeted therapy initiated, presence of brain metastasis and presence of ulcerations at targeted therapy initiation. These characteristics were described in terms of mean ± SD (median) for quantitative variables and in terms of number (%) for qualitative variables. Quantitative characteristics were categorized if needed.

### Statistical analysis

mRNA expression and CNVs for the studied genes were collected at baseline and at relapse observation. Copy number variations were dichotomized as amplifications (level >5 copy number variations) and as deletions (level < 0.5 copy number variations). Quantitative mRNA expression data were converted to LOG_2_ in order to normalize their distribution.

Two time to event endpoints were defined: Progression free survival (PFS) and overall survival (OS) defined as time between targeted therapy initiation and disease progression and death respectively. To detect disease progression related to BRAF inhibitors, patients were censored at the BRAF inhibitor interruption for PFS. As death occurred mainly after BRAF inhibitor interruption, patients were censored at the last date of follow-up for OS. A third binary outcome was defined as the best observed response to targeted therapy: Response (complete or partial) or no response (stable disease or progressive disease).

Associations of clinical baseline variables with time to event endpoints (PFS and OS) were expressed as Hazard ratio (HR) and their 95% confidence interval (95% CI).

The influence of DNA alterations on duration of response was studied in terms of number of alterations (quantitative variable). A binary variable was also constructed: At least one DNA alteration (CNVs or mutations) *vs* No DNA alteration. Univariate analysis with OS and PFS was performed and adjustment with relevant clinical baseline characteristics was performed in a Cox multivariate model. These clinical baseline characteristics were selected among the candidate variables (*p*-value <0.20) according to the minimization of the Akaike information criterion (AIC).

To examine the relevance of a mRNA expression profile as a prognostic information for OS and PFS, the supervised principal components analysis described by Bair *et al*. [55] was conducted to isolate a subset of genes correlated with survival. This method has been widely used in several studies to identify predictive gene signatures [56–58]. All genes are tested one by one in univariate analysis and are assigned a Cox score representative of their degree of association with PFS and OS. Genes with the highest absolute Cox scores (*i.e.* high correlation with survival) were selected for each endpoint and allowed us to compute a PFS gene signature and OS gene signature. Cross validation was used to determine the optimal threshold for Cox scores and to select the most significant genes to include in gene signatures. The first two principal components of these genes’ subsets were then computed in a Cox multivariate model. To assess the interest of such a gene signature, risk prediction was calculated using 3 multivariate models: 1) the clinical baseline characteristics selected according to the minimization of the AIC criterion only 2) the gene expression profile only and 3) the gene expression profile adjusted on relevant clinical baseline characteristics selected according to the minimization of the AIC criterion. Patients were categorized as low risk or high risk according to the median risk prediction of each model. PFS and OS were estimated with the Kaplan Meier method and differences between high and low risk group were assessed with the log rank test.

### External validation

To assess external relevance of our findings, literature was screened to select studies with genomic analysis in metastatic melanoma patients treated with a BRAF inhibitor as monotherapy. We focused on set of patients with gene expression data at baseline (prior to BRAF inhibitor initiation) and with available clinical course (Progression Free survival and/or Overall survival). Gene set enrichment analysis (GSEA) was then performed on the selected external dataset. GSEA was run on 2 *a priori* set of genes: 1) Set 2: PFS gene signature; 2) Set 3: OS gene signature.

Each patient of the dataset was assigned a phenotype label and two approaches were conducted: 1) Continuous labelling: Patients were defined by their PFS; 2) Categorical labelling: Patients were ranked according to their PFS and categorized into two groups according to the median PFS: Good *vs* Bad responders. Metric used for GSEA were Pearson coefficient and ratio of classes for continuous and categorical labelling respectively.

## SUPPLEMENTARY MATERIALS FIGURES AND TABLES






