# Supplementary material for: A targeted genomic alteration analysis predicts survival of melanoma patients under BRAF inhibitors
Source: Oncotarget. 2019 Mar 1;10(18):1669–87. doi: 10.18632/oncotarget.26707 (PMC6422198; doi:10.18632/oncotarget.26707)
Supplement: Supplementary file 1 [file oncotarget-10-1669-s001.pdf]

A targeted genomic alteration analysis predicts survival of melanoma patients under BRAF inhibitors

SUPPLEMENTARY MATERIALS

REFERENCES

1. Poulikakos PI, Persaud Y, Janakiraman M, Kong X, Ng C, Moriceau G, Shi H, Atefi M, Titz B, Gabay MT, Salton M, Dahlman KB, Tadi M, et al. RAF inhibitor resistance is mediated by dimerization of aberrantly spliced BRAF(V600E). Nature. 2011; 480:387–90. https://doi.org/10.1038/nature10662.
2. Shi H, Moriceau G, Kong X, Lee MK, Lee H, Koya RC, Ng C, Chodon T, Scolyer RA, Dahlman KB, Sosman JA, Kefford RF, Long GV, et al. Melanoma whole-exome sequencing identifies (V600E)B-RAF amplification-mediated acquired B-RAF inhibitor resistance. Nat Commun. 2012; 3:724. https://doi.org/10.1038/ncomms1727.
3. Shi H, Hugo W, Kong X, Hong A, Koya RC, Moriceau G, Chodon T, Guo R, Johnson DB, Dahlman KB, Kelley MC, Kefford RF, Chmielowski B, et al. Acquired resistance and clonal evolution in melanoma during BRAF inhibitor therapy. Cancer Discov. 2014; 4:80–93. https://doi.org/10.1158/2159-8290.CD-13-0642.
4. Rizos H, Menzies AM, Pupo GM, Carlino MS, Fung C, Hyman J, Haydu LE, Mijatov B, Becker TM, Boyd SC, Howle J, Saw R, Thompson JF, et al. BRAF inhibitor resistance mechanisms in metastatic melanoma: spectrum and clinical impact. Clin Cancer Res. 2014; 20:1965–77. https://doi.org/10.1158/1078-0432.CCR-13-3122.
5. KEGG_pathwaysInCancer. Available from: http://www.genome.jp/kegg-bin/show_pathway?hsa05200.
6. FoundationOne. Available from: https://www.foundationmedicineasia.com/dam/assets/pdf/FOne_Current_Gene_List.pdf.
7. Hodis E, Watson IR, Kryukov GV, Arold ST, Imielinski M, Theurillat JP, Nickerson E, Auclair D, Li L, Place C, Dicara D, Ramos AH, Lawrence MS, et al. A landscape of driver mutations in melanoma. Cell. 2012; 150:251–63. https://doi.org/10.1016/j.cell.2012.06.024.
8. Kandoth C, McLellan MD, Vandin F, Ye K, Niu B, Lu C, Xie M, Zhang Q, McMichael JF, Wyczalkowski MA, Leiserson MDM, Miller CA, Welch JS, et al. Mutational landscape and significance across 12 major cancer types. Nature. 2013; 502:333–339. https://doi.org/10.1038/nature12634.
9. Lawrence MS, Stojanov P, Mermel CH, Robinson JT, Garraway LA, Golub TR, Meyerson M, Gabriel SB, Lander ES, Getz G. Discovery and saturation analysis of cancer genes across 21 tumour types. Nature. 2014; 505:495–501. https://doi.org/10.1038/nature12912.
10. Nazarian R, Shi H, Wang Q, Kong X, Koya RC, Lee H, Chen Z, Lee MK, Attar N, Sazegar H, Chodon T, Nelson SF, McArthur G, et al. Melanomas acquire resistance to B-RAF(V600E) inhibition by RTK or N-RAS upregulation. Nature. 2010; 468:973–77. https://doi.org/10.1038/nature09626.
11. Johnson DB, Menzies AM, Zimmer L, Eroglu Z, Ye F, Zhao S, Rizos H, Sucker A, Scolyer RA, Gutzmer R, Gogas H, Kefford RF, Thompson JF, et al. Acquired BRAF inhibitor resistance: A multicenter meta-analysis of the spectrum and frequencies, clinical behaviour, and phenotypic associations of resistance mechanisms. Eur J Cancer. 2015; 51:2792–99. https://doi.org/10.1016/j.ejca.2015.08.022.
12. Wagle N, Emery C, Berger MF, Davis MJ, Sawyer A, Pochanard P, Kehoe SM, Johannessen CM, Macconaill LE, Hahn WC, Meyerson M, Garraway LA. Dissecting therapeutic resistance to RAF inhibition in melanoma by tumor genomic profiling. J Clin Oncol. 2011; 29:3085–96. https://doi.org/10.1200/JCO.2010.33.2312.
13. Johannessen CM, Boehm JS, Kim SY, Thomas SR, Wardwell L, Johnson LA, Emery CM, Stransky N, Cogdill AP, Barretina J, Caponigro G, Hieronymus H, Murray RR, et al. COT drives resistance to RAF inhibition through MAP kinase pathway reactivation. Nature. 2010; 468:968–72. https://doi.org/10.1038/nature09627.
14. Johannessen CM, Johnson LA, Piccioni F, Townes A, Frederick DT, Donahue MK, Narayan R, Flaherty KT, Wargo JA, Root DE, Garraway LA. A melanocyte lineage program confers resistance to MAP kinase pathway inhibition. Nature. 2013; 504:138–42. https://doi.org/10.1038/nature12688.
15. Montagut C, Sharma SV, Shioda T, McDermott U, Ulman M, Ulkus LE, Dias-Santagata D, Stubbs H, Lee DY, Singh A, Drew L, Haber DA, Settleman J. Elevated CRAF as a potential mechanism of acquired resistance to BRAF inhibition in melanoma. Cancer Res. 2008; 68:4853–61. https://doi.org/10.1158/0008-5472.CAN-07-6787.

16. Villanueva J, Vultur A, Lee JT, Somasundaram R, Fukunaga-Kalabis M, Cipolla AK, Wubbenhorst B, Xu X, Gimotty PA, Kee D, Santiago-Walker AE, Letrero R, D'Andrea K, et al. Acquired resistance to BRAF inhibitors mediated by a RAF kinase switch in melanoma can be overcome by cotargeting MEK and IGF-1R/PI3K. Cancer Cell. 2010; 18:683–95. https://doi.org/10.1016/j.ccr.2010.11.023.

17. Vergani E, Vallacchi V, Frigerio S, Deho P, Mondellini P, Perego P, Cassinelli G, Lanzi C, Testi MA, Rivoltini L, Bongarzone I, Rodolfo M. Identification of MET and SRC activation in melanoma cell lines showing primary resistance to PLX4032. Neoplasia. 2011; 13:1132–42. https://doi.org/10.1593/neo.111102.

18. Straussman R, Morikawa T, Shee K, Barzily-Rokni M, Qian ZR, Du J, Davis A, Mongare MM, Gould J, Frederick DT, Cooper ZA, Chapman PB, Solit DB, et al. Tumour micro-environment elicits innate resistance to RAF inhibitors through HGF secretion. Nature. 2012; 487:500–04. https://doi.org/10.1038/nature11183.

19. Wilson TR, Fridlyand J, Yan Y, Penuel E, Burton L, Chan E, Peng J, Lin E, Wang Y, Sosman J, Ribas A, Li J, Moffat J, et al. Widespread potential for growth-factor-driven resistance to anticancer kinase inhibitors. Nature. 2012; 487:505–09. https://doi.org/10.1038/nature11249.

20. Sun C, Wang L, Huang S, Heynen GJ, Prahallad A, Robert C, Haanen J, Blank C, Wesseling J, Willems SM, Zecchin D, Hobor S, Bajpe PK, et al. Reversible and adaptive resistance to BRAF(V600E) inhibition in melanoma. Nature. 2014; 508:118–22. https://doi.org/10.1038/nature13121.

21. Girotti MR, Pedersen M, Sanchez-Laorden B, Viros A, Turajlic S, Niculescu-Duvaz D, Zambon A, Sinclair J, Hayes A, Gore M, Lorigan P, Springer C, Larkin J, et al. Inhibiting EGF receptor or SRC family kinase signaling overcomes BRAF inhibitor resistance in melanoma. Cancer Discov. 2013; 3:158–67. https://doi.org/10.1158/2159-8290.CD-12-0386.

22. Sinnberg T, Makino E, Krueger MA, Velic A, Macek B, Rothbauer U, Groll N, Pötz O, Czemmel S, Niessner H, Meier F, Ikenberg K, Garbe C, Schittek B. A Nexus Consisting of Beta-Catenin and Stat3 Attenuates BRAF Inhibitor Efficacy and Mediates Acquired Resistance to Vemurafenib. EBioMedicine. 2016; 8:132–49. https://doi.org/10.1016/j.ebiom.2016.04.037.

23. Smalley KS, Lioni M, Dalla Palma M, Xiao M, Desai B, Egyhazi S, Hansson J, Wu H, King AJ, Van Belle P, Elder DE, Flaherty KT, Herlyn M, Nathanson KL. Increased cyclin D1 expression can mediate BRAF inhibitor resistance in BRAF V600E-mutated melanomas. Mol Cancer Ther. 2008; 7:2876–83. https://doi.org/10.1158/1535-7163.MCT-08-0431.

24. Nathanson KL, Martin AM, Wubbenhorst B, Greshock J, Letrero R, D'Andrea K, O'Day S, Infante JR, Falchook GS, Arkenau HT, Millward M, Brown MP, Pavlick A, et al. Tumor genetic analyses of patients with metastatic melanoma treated with the BRAF inhibitor dabrafenib (GSK2118436). Clin Cancer Res. 2013; 19:4868–78. https://doi.org/10.1158/1078-0432.CCR-13-0827.

25. Wei WJ, Sun ZK, Shen CT, Song HJ, Zhang XY, Qiu ZL, Luo QY. Obatoclax and LY3009120 Efficiently Overcome Vemurafenib Resistance in Differentiated Thyroid Cancer. Theranostics. 2017; 7:987–1001. https://doi.org/10.7150/thno.17322.

26. Haq R, Yokoyama S, Hawryluk EB, Jönsson GB, Frederick DT, McHenry K, Porter D, Tran TN, Love KT, Langer R, Anderson DG, Garraway LA, Duncan LM, et al. BCL2A1 is a lineage-specific antiapoptotic melanoma oncogene that confers resistance to BRAF inhibition. Proc Natl Acad Sci U S A. 2013; 110:4321–26. https://doi.org/10.1073/pnas.1205575110.

27. Shao Y, Aplin AE. Akt3-mediated resistance to apoptosis in B-RAF-targeted melanoma cells. Cancer Res. 2010; 70:6670–81. https://doi.org/10.1158/0008-5472.CAN-09-4471.

28. Lai F, Jiang CC, Farrelly ML, Zhang XD, Hersey P. Evidence for upregulation of Bim and the splicing factor SRp55 in melanoma cells from patients treated with selective BRAF inhibitors. Melanoma Res. 2012; 22:244–51. https://doi.org/10.1097/CMR.0b013e328353eff2.

29. Fatkhutdinov N, Sproesser K, Krepler C, Liu Q, Brafford PA, Herlyn M, Aird KM, Zhang R. Targeting RRM2 and Mutant BRAF Is a Novel Combinatorial Strategy for Melanoma. Mol Cancer Res. 2016; 14:767–75. https://doi.org/10.1158/1541-7786.MCR-16-0099.

30. Paraiso KH, Xiang Y, Rebecca VW, Abel EV, Chen YA, Munko AC, Wood E, Fedorenko IV, Sondak VK, Anderson AR, Ribas A, Palma MD, Nathanson KL, et al. PTEN loss confers BRAF inhibitor resistance to melanoma cells through the suppression of BIM expression. Cancer Res. 2011; 71:2750–60. https://doi.org/10.1158/0008-5472.CAN-10-2954.

31. Trunzer K, Pavlick AC, Schuchter L, Gonzalez R, McArthur GA, Hutson TE, Moschos SJ, Flaherty KT, Kim KB, Weber JS, Hersey P, Long GV, Lawrence D, et al. Pharmacodynamic effects and mechanisms of resistance to vemurafenib in patients with metastatic melanoma. J Clin Oncol. 2013; 31:1767–74. https://doi.org/10.1200/JCO.2012.44.7888.

Supplementary Table 1: Univariate analysis of clinical baseline characteristics for progression free survival and overall survival

	Progression free survival		Overall survival	
	HR [95% CI] (Univariate analysis)	*P*-value	HR [95% CI] (Univariate analysis)	*P*-value
**Age at therapy initiation > 65 years**	1.29 [0.74; 2.24]	0.371	1.09 [0.63; 1.89]	0.757
**Male sex**	2.27 [1.25; 4.13]	0.006	1.79 [1.02; 3.13]	0.041
**Melanoma subtype**				
Nodular	ref	0.355	ref	0.063
Superficial spreading melanoma	0.66 [0.36; 1.19]		0.52 [0.28; 0.94]	
Others or undertermined	0.86 [0.40; 1.85]		0.48 [0.21; 1.12]	
**Breslow thickness > 2.5 mm**	1.04 [0.59; 1.82]	0.897	1.35 [0.77; 2.38]	0.297
**Ulceration**	2.95 [1.51; 5.73]	0.001	1.84 [1.01; 3.37]	0.044
**Stage**				
III	ref	0.043	ref	0.126
IV	2.00 [1.01; 3.96]		1.70 [0.86; 3.38]	
**Brain metastasis**	3.84 [2.02; 7.31]	<0.001	3.72 [2.06; 6.72]	<0.001
**First BRAF inhibitor initiated**				
Vemurafenib	ref	0.354	ref	0.049
Dabrafenib	0.65 [0.26; 1.63]		0.33 [0.10; 1.06]	

P-values are calculated using the log-rank test. Bold *P*-values indicate candidate variables for further adjustment (*P*-value ≤ 0.20). HR, Hazard Ratio; CI, Confidence interval, mm, millimeters.

Supplementary Table 2: List of studied genes and their relevance in BRAF inhibitor resistance. See Supplementary_Table_2

Supplementary Table 3: Data obtained from DNA and mRNA analysis for every samples (Baseline and relapse-matched samples). DNA alterations are expressed as absolute copy numbers and mRNA expression as the ratio 'copy number of gene of interest/copy number of *PPIA*'. *indicates that macrodissection was performed when <50% tumor cells. (VAF: Variant allele frequency). See Supplementary_Table_3

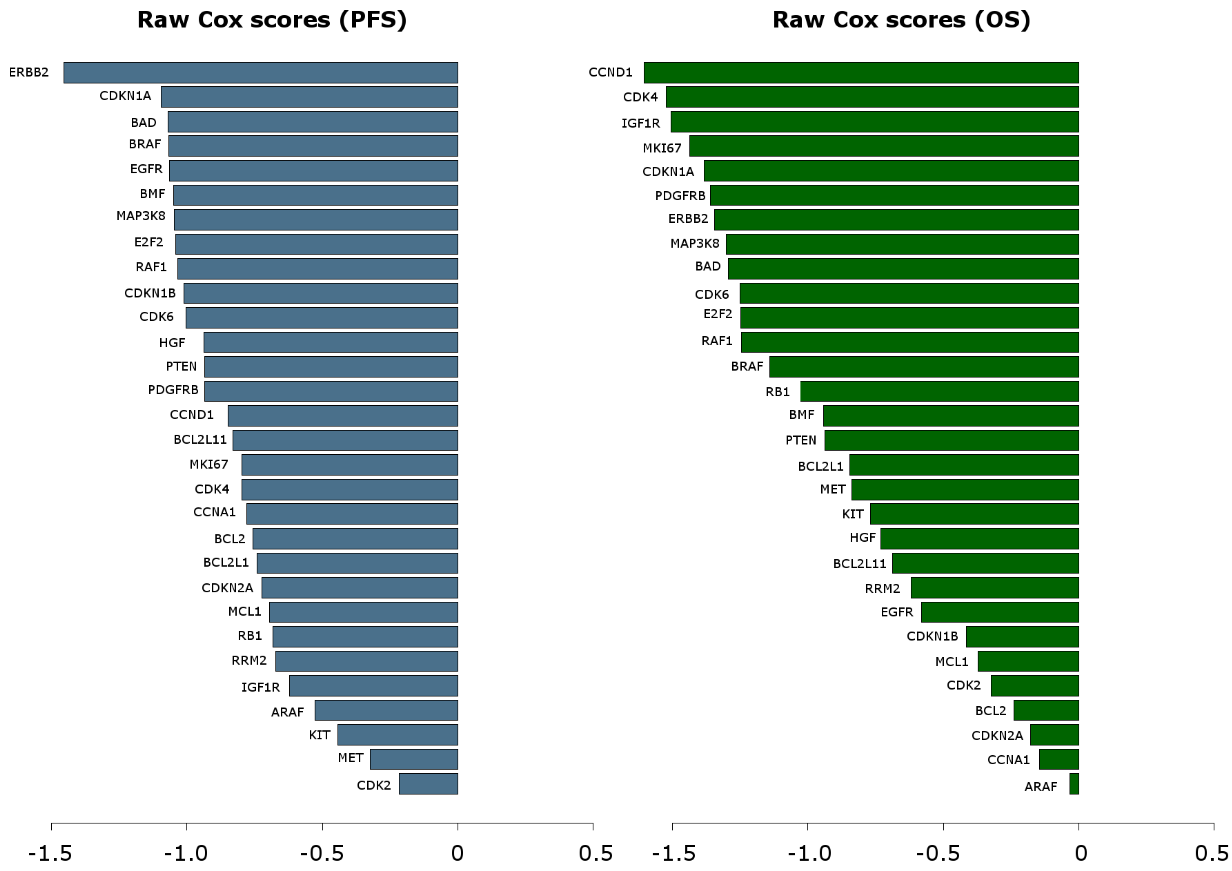

Supplementary Figure 1: Raw Cox scores of each gene obtained for PFS and OS from the supervised principal components analysis. Negative scores indicate a protective association with survival. The lower the score is, the higher the protective association.

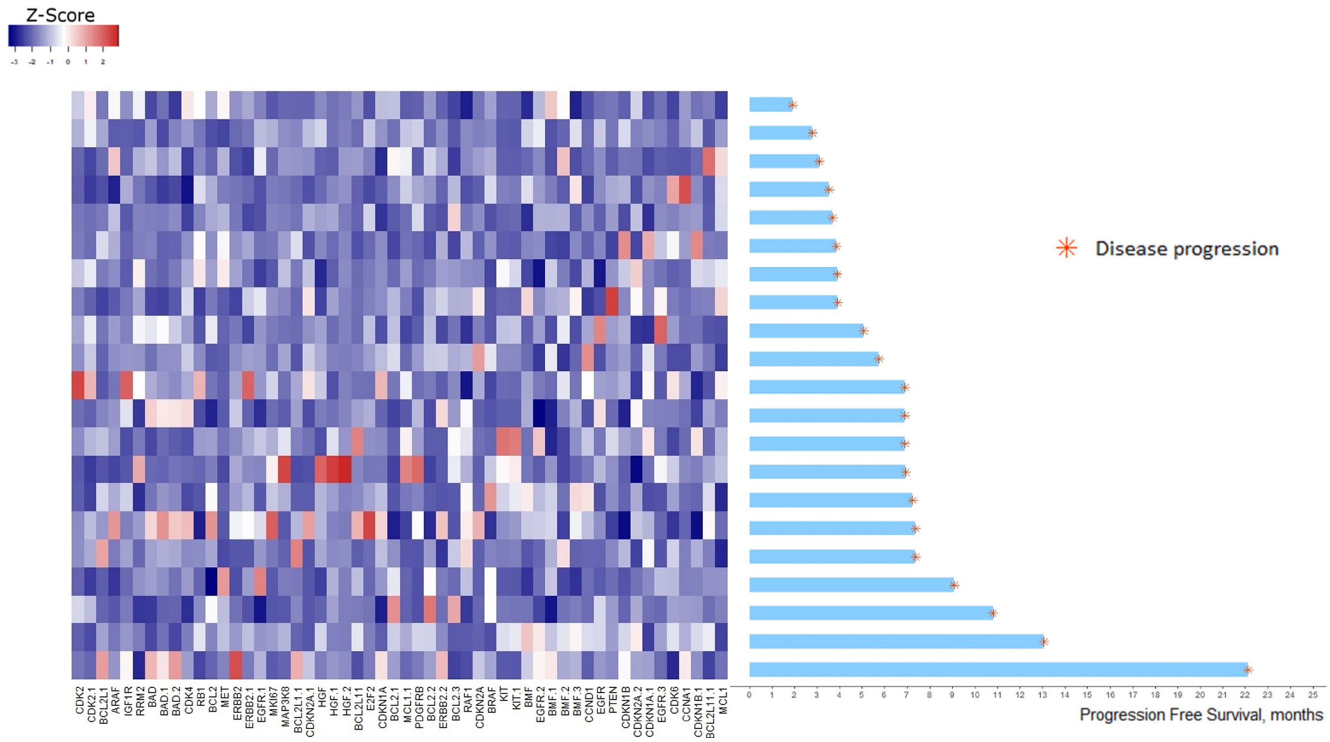

Supplementary Figure 2: Heatmaps of baseline mRNA expression for the 21 patients of the Rizos *et al.* [4] dataset. Color represents the relative expression of each gene in each sample, centered on the mean and scaled to the standard deviation. Blue is low expression and red is high expression. Patients are ranked according to their progression free survival. mRNA expression was obtained from Illumina HumanHT-12 V4.0 expression beadchip and all *loci* corresponding to our genes of interest were analyzed.

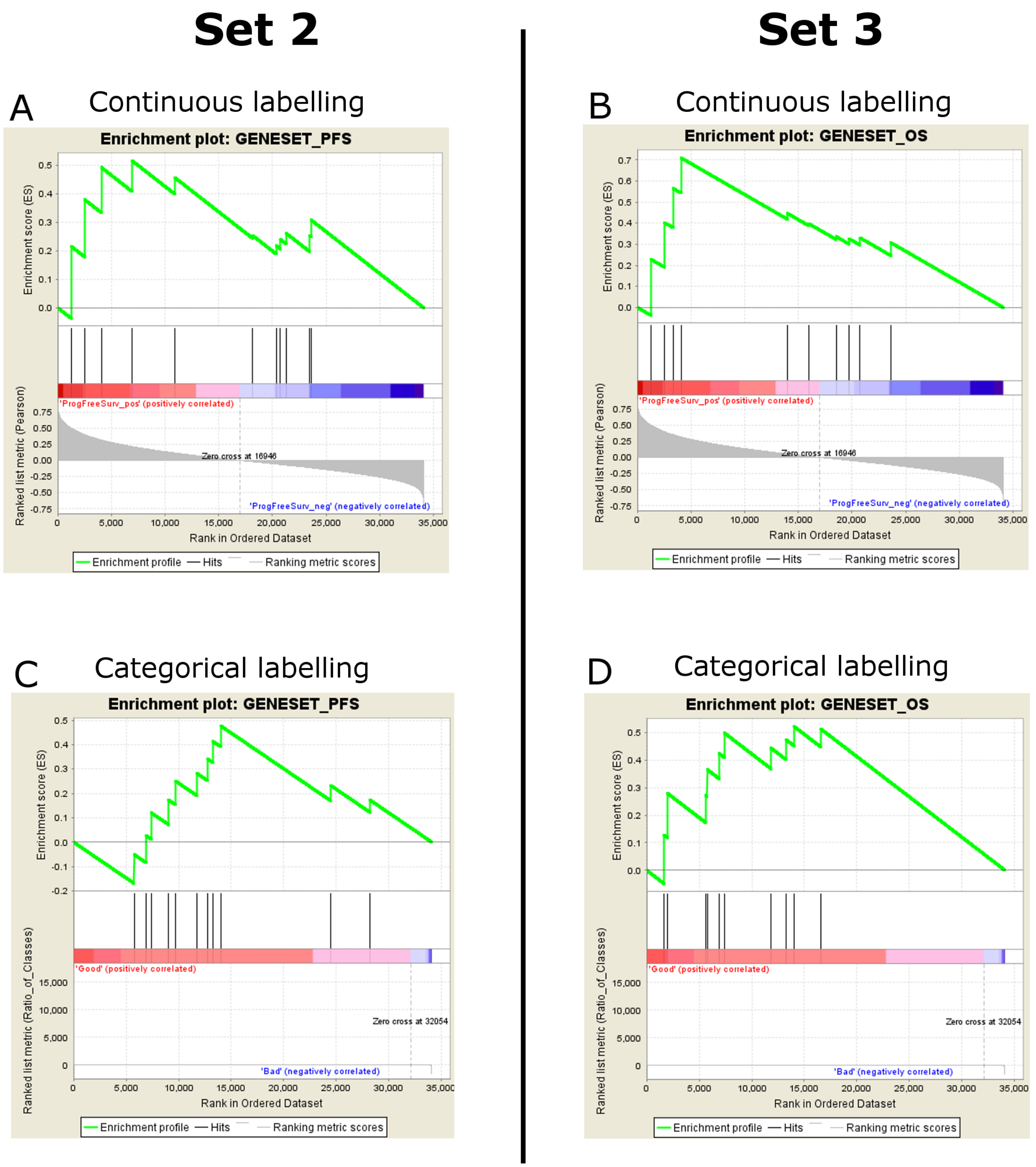

Supplementary Figure 3: Enrichment plots for the gene set enrichment analysis performed on Rizos *et al.* [4] dataset. (**A**) Enrichment plot for set 2 (PFS gene signature) with patient phenotypes defined as their corresponding PFS (continuous labelling). (**B**) Enrichment plot for set 3 (OS gene signature) with patient phenotypes defined as their corresponding PFS (continuous labelling). (**C**) Enrichment plot for set 2 (PFS gene signature) with patient phenotypes defined as good or bad responders (categorical labelling). (**D**) Enrichment plot for set 3 (OS gene signature) with patient phenotypes defined as good or bad responders (categorical labelling).

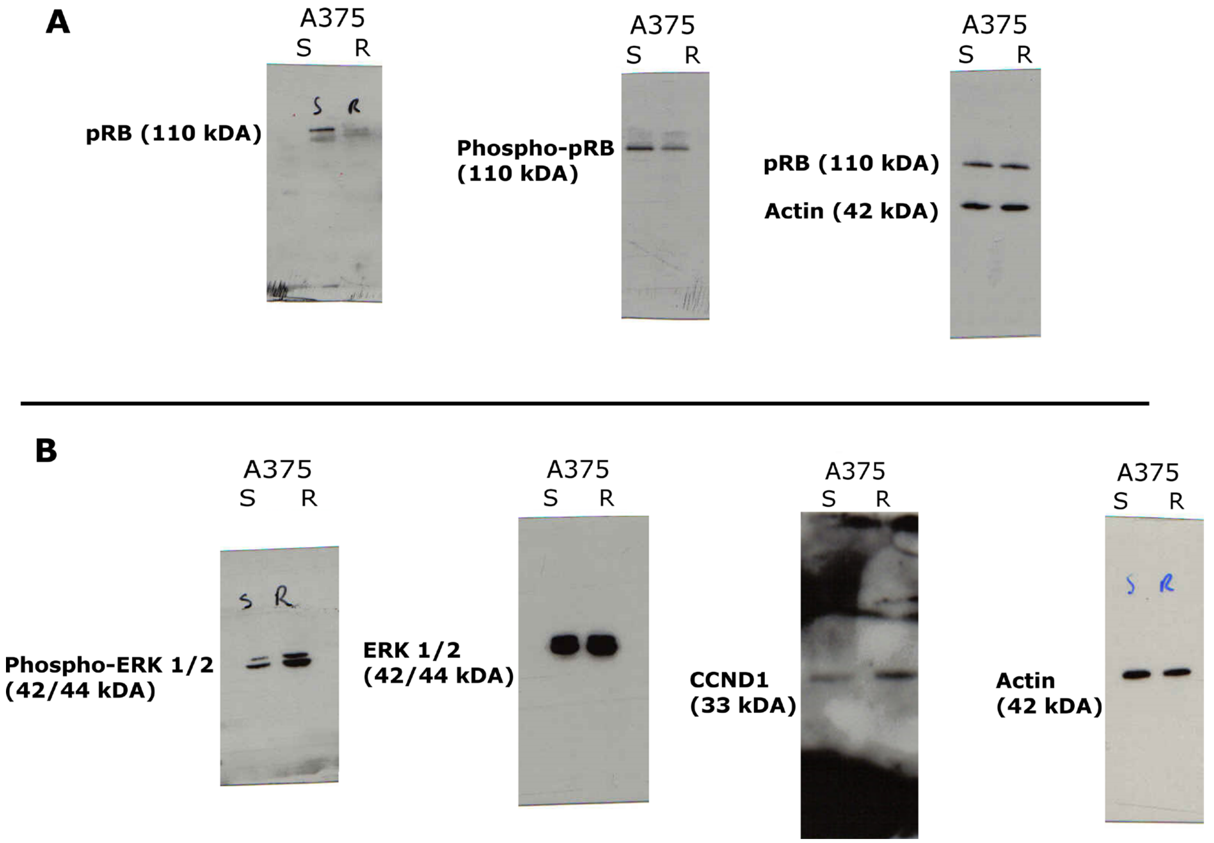

Supplementary Figure 4: Uncropped blots (**A**) Uncropped blots of the analysis of the levels of phospho-pRB, pRB and actin in the vemurafenib sensitive parental A375 cells (A375-S) and vemurafenib resistant A375 cells (A375-R). Actin was used as internal control. Representative blots of three independent experiments are shown. (**B**) Uncropped blots of the levels of phospho-ERK, ERK and CCND1 in the vemurafenib sensitive parental A375 cells and vemurafenib resistant A375-R cells following 3 months with 1 μM of vemurafenib. Actin was used as internal control. Representative blots of three independent experiments are shown.

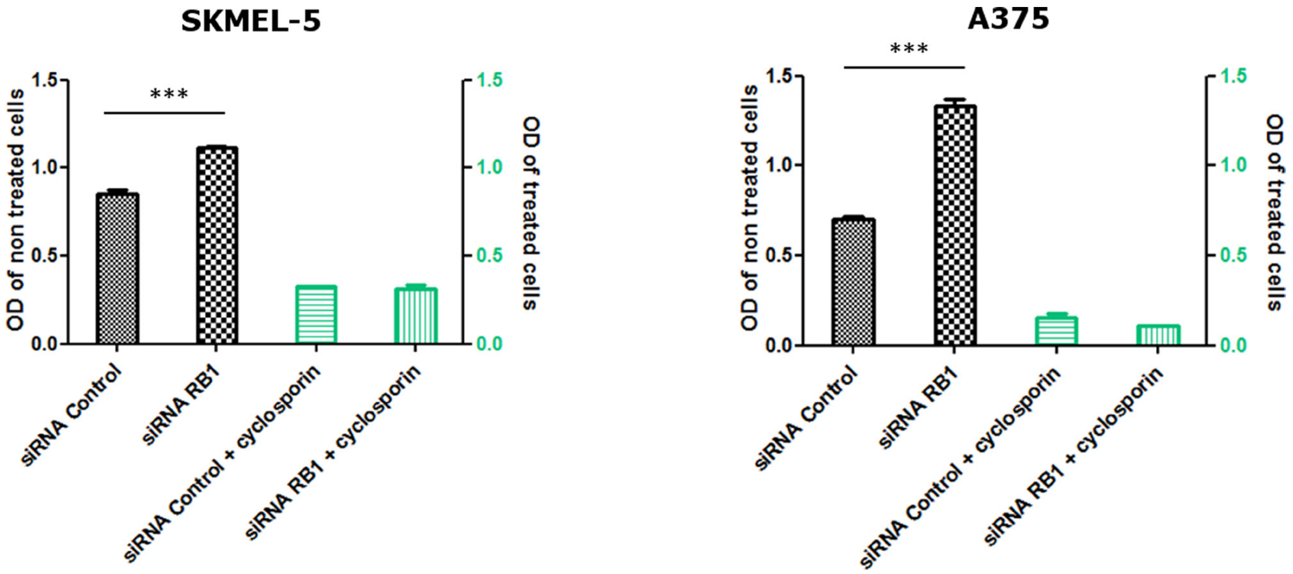

Supplementary Figure 5: Proliferation assay in SKMEL-5 and A375 cell lines undergoing a *RB1* downregulation with RNA interference and treated or not with cyclosporin. Bars represent means from three independent experiments. $^{***}p < 0.0001$ (siRNA Control: small interfering RNA control; siRNA RB1: small interfering RNA RB1; OD: Optical density).

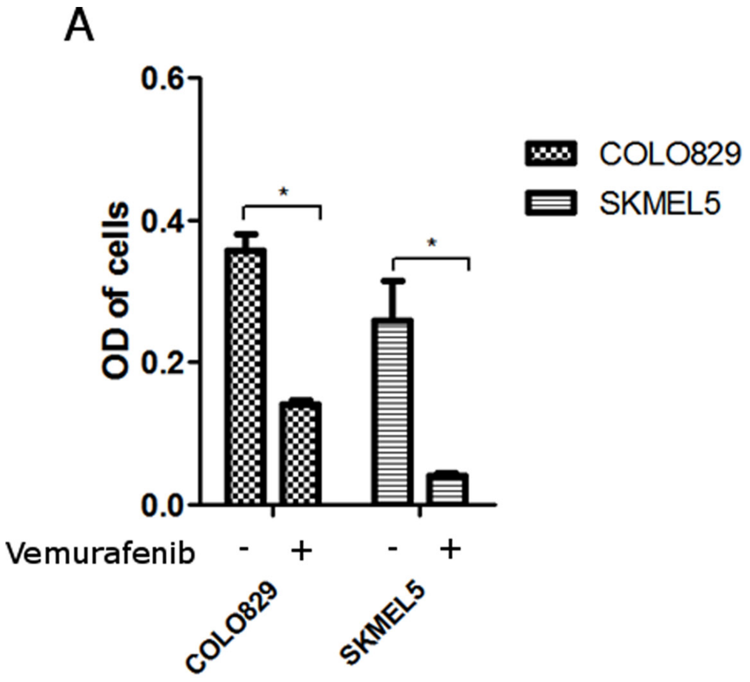

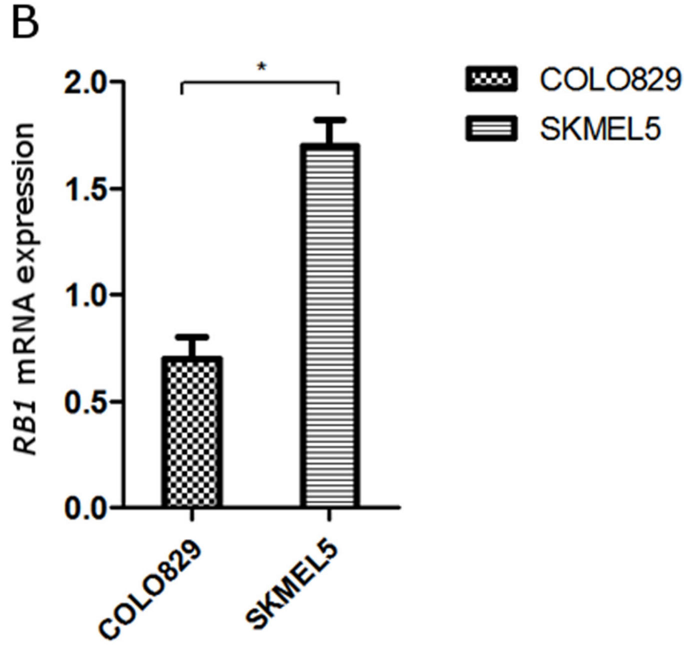

Supplementary Figure 6: Comparison of *RB1* mRNA expression and proliferation rate in 2 melanoma cell lines. (**A**) Proliferation assay in SKMEL-5 and COLO829 cell lines treated or not with vemurafenib 1 µM for 5 days. (**B**) Levels of *RB1* mRNA expression in SKMEL-5 and COLO829 cell lines assessed in vemurafenib treated cell lines at day 3. TATA box binding protein was used as housekeeping gene for normalization. Bars represent means from three independent experiments. $^{*}p < 0.01$ (OD: Optical density).

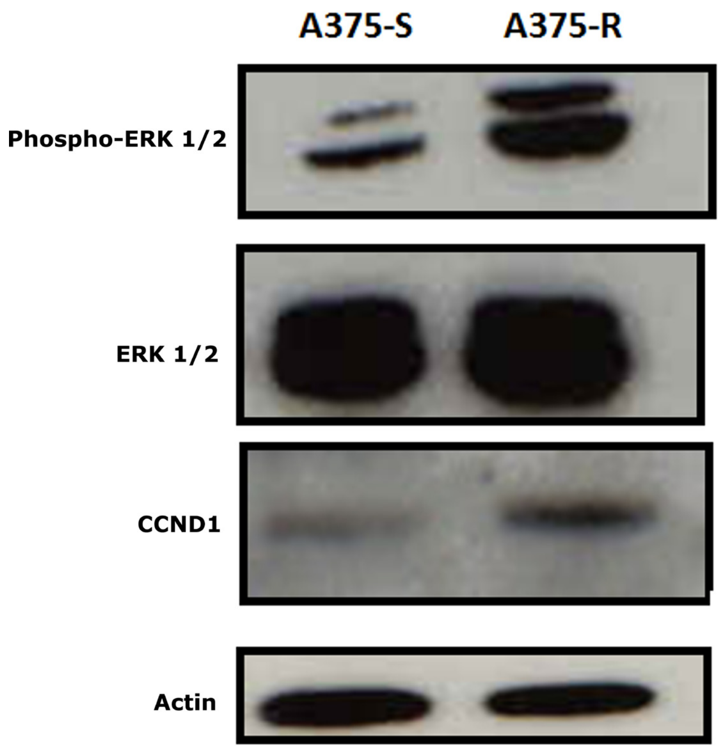

Supplementary Figure 7: Comparison of ERK activation level in vemurafenib resistant A375 melanoma cells (A375-R) to parental A375 cells (A375-S). Western blot analysis of the levels of phospho-ERK, ERK and CCND1 in the vemurafenib sensitive parental A375 cells and vemurafenib resistant A375-R cells following 3 months with 1 μM of vemurafenib. Actin was used as internal control. Representative blots of three independent experiments are shown.
