# Supplementary material for: A targeted genomic alteration analysis predicts survival of melanoma patients under BRAF inhibitors
Source: Oncotarget. 2019 Mar 1;10(18):1669–87. doi: 10.18632/oncotarget.26707 (PMC6422198; doi:10.18632/oncotarget.26707)
Supplement: Supplementary file 2 [file oncotarget-10-1669-s002.docx]

**Supplementary Table 2: List of studied genes and their relevance in BRAF inhibitor resistance**

| **Pathway** | **Gene** | **Type of analysis** | | **Relevance in BRAF inhibitor resistance** | **Reference/Datasource list** |
| --- | --- | --- | --- | --- | --- |
|  |  | mRNA expression | Mutation /copy number analysis |  |  |
| **MAPK Pathway related genes** | *BRAF* | X  and splice variants | X | -BRAF splice variants associated with acquired resistance to vemurafenib.  -BRAF overexpression leads to ERK reactivation.  -BRAF amplification associated with resistance in patients treated with BRAF inhibitors. | Poulikakos *et al.* (2011) [1]; Shi *et al.* (2012) [2]; Shi *et al.* (2014) [3]; Rizos *et al.* (2014) [4]/KEGG_pathwaysInCancer [5]; FoundationOne [6]; LyndaChin-Cell2012 [7]; PanCancer_12Cancer_SignifMutatedGenes_Nature 2013 [8]; SaturationAnalysisOn21CancerTypes [9] |
|  | *NRAS* |  | X | -NRAS mutations reported in relapse tumors of melanoma patients treated with BRAF inhibitors.  -NRAS mutations detected in a subset of tumors patients prior to BRAF inhibitor treatment.  -Increase of NRAS gDNA copy numbers described in resistant tumors compared to baseline tumors in melanoma patients treated with vemurafenib. | Nazarian *et al.* (2010) [10]; Rizos *et al.* (2014) [4]; Shi *et al.* (2014) [3]; Johnson *et al.* (2015) [11]/KEGG_pathwaysInCancer [5]; FoundationOne [6]; LyndaChin-Cell2012 [7]; PanCancer_12Cancer_SignifMutatedGenes_Nature  2013 [8]; SaturationAnalysisOn21CancerTypes [9] |
|  | *MAP2K1* |  | X | -MAP2K1 mutations reported in progressive tumors of melanoma patients treated with BRAF inhibitors. | Rizos *et al.* (2014) [4]; Shi *et al.* (2014) [3]; Wagle *et al.* (2011) [12]/KEGG_pathwaysInCancer [5]; FoundationOne [6]; LyndaChin-Cell2012 [7]; SaturationAnalysisOn21CancerTypes [9] |
|  | *MAP3K8* | X |  | -Overexpression of MAP3K8 described as a resistance mechanism in melanoma treated with BRAF inhibitor (vemurafenib) | Johannessen et al. (2010) [13]/ORF-MAPKiResistance [14] |
|  | *RAF1* | X |  | -Overexpression of RAF1 reported as a resistance mechanism in melanoma treated with a BRAF inhibitor | Montagut *et al.* (2008) [15]; Villanueva *et al.* (2010) [16]; Johannessen *et al.* (2010) [13]/KEGG_pathwaysInCancer [5]; FoundationOne [6]; ORF-MAPKiResistance [14] |
|  | *ARAF* | X |  | -Acquired resistance to BRAF inhibitors mediated by a RAF kinase switch in melanoma can be overcome by co-targeting MEK and IGF-1R/PI3K | Villanueva *et al.* (2010) [16]/KEGG_pathwaysInCancer [5]; FoundationOne [6] |
| **Tyrosine kinase receptors related genes** | *PDGFRB* | X |  | -Melanomas acquire resistance to BRAF(V600E) inhibition through RTK or NRAS upregulation  -Overexpression of PDGFRB in relapsed tumors compared to baseline tumors of melanoma patients treated with vemurafenib | Nazarian *et al.* (2010) [10]/KEGG_pathwaysInCancer [5]; FoundationOne [6] |
|  | *IGF1R* | X |  | -Acquired resistance to BRAF inhibitors mediated by a RAF kinase switch in melanoma can be overcome by co-targeting MEK and IGF-1R/PI3K.  -IGF-1R/PI3K signaling was enhanced in resistant melanomas. | Villanueva *et al.* (2010) [16]/KEGG_pathwaysInCancer [5]; FoundationOne [6] |
|  | *MET* | X | X | -Identification of MET activation in melanoma cell lines showing primary resistance to vemurafenib.  -Overexpression of MET described in melanoma cell lines resistant to vemurafenib. | Vergani *et al.* (2011) [17]; Nazarian *et al.* (2010) [10]/KEGG_pathwaysInCancer [5]; FoundationOne [6]; SaturationAnalysisOn21CancerTypes [9] |
|  | *HGF* | X |  | -Tumor microenvironment elicits innate resistance to RAF inhibitors through HGF secretion.  -HGF expression confers resistance to vemurafenib in BRAF mutant melanoma cells | Straussman *et al.* (2012) [18]; Wilson *et al.* (2012) [19]/KEGG_pathwaysInCancer [5]; FoundationOne [6]; PanCancer_12Cancer_SignifMutatedGenes_Nature2013 [8] |
|  | *KIT* | X |  | -Overexpression of KIT described in melanoma cell lines resistant to vemurafenib. | Nazarian *et al.* (2010) [10]/KEGG_pathwaysInCancer [5];  FoundationOne [6]; LyndaChin-Cell2012 [7];  PanCancer_12Cancer_SignifMutatedGenes_Nature  2013 [8]; SaturationAnalysisOn21CancerTypes [9] |
|  | *EGFR* | X |  | -Upregulation of EGFR described in melanoma cell lines resistant to vemurafenib.  -Overexpression of EGFR reported in melanoma tumors after the development of resistance to BRAF or MEK inhibitors. | Nazarian *et al.* (2010) [10]; Sun *et al.* (2014) [20]; Girotti *et al.* (2013) [21]/KEGG_pathwaysInCancer [5]; FoundationOne [6]; PanCancer_12Cancer_SignifMutatedGenes_Nature2013 [8];  SaturationAnalysisOn21CancerTypes [9] |
|  | *ERBB2* | X |  | -Overexpression of ERBB2 described in relapsed tumors compared to baseline tumors of melanoma patients treated with vemurafenib | Johannessen *et al.* (2010) [13]/KEGG_pathwaysInCancer [5];  FoundationOne [6]; SaturationAnalysisOn21CancerTypes [9] |
| **Cell cycle related genes** | *CTNNB1* |  | X | -Amplification of CTNNB1 described in two melanoma cell lines resistant to vemurafenib.  -A nexus consisting of Beta-catenin and Stat3 attenuates RAF inhibitor efficacy and mediates acquired resistance to vemurafenib. | Vergani *et al.* (2011) [17]; Sinnberg *et al.* (2016) [22]/KEGG_pathwaysInCancer [5]; FoundationOne [6]; LyndaChin-Cell2012 [7]; PanCancer_12Cancer_SignifMutatedGenes_Nature  2013 [8]; SaturationAnalysisOn21CancerTypes [9] |
|  | *MKI67* | X |  | -Overexpression of MKI67 described in progressive tumors compared to baseline tumors of melanoma patients treated with BRAF inhibitors. | Shi *et al.* (2014) [3] |
|  | *CDK4* | X | X | -CDK4 overexpression/mutation contributes to BRAF inhibitor resistance in melanoma cell lines. | Smalley *et al.* (2008) [23]/KEGG_pathwaysInCancer [5]; FoundationOne [6]; LyndaChin-Cell2012 [7]; SaturationAnalysisOn21CancerTypes [9] |
|  | *E2F2* | X |  | -Cell cycle activation mediates resistance to BRAF inhibitors. | KEGG_pathwaysInCancer [5] |
|  | *RB1* | X | X | -Cell cycle activation mediates resistance to BRAF inhibitors. | KEGG_pathwaysInCancer [5]; FoundationOne [6]; PanCancer_12Cancer_SignifMutatedGenes_Nature2013 [8]; SaturationAnalysisOn21CancerTypes [9] |
|  | *CDK2* | X |  | -Cell cycle activation mediates resistance to BRAF inhibitors. | KEGG_pathwaysInCancer [5] |
|  | *CDK6* | X | X | -Cell cycle activation mediates resistance to BRAF inhibitors. | KEGG_pathwaysInCancer [5]; FoundationOne [6] |
|  | *CCND1* | X | X | -CCND1 overexpression contributes to BRAF inhibitor resistance in melanoma cell lines.  -CCND1 amplification in melanoma tumors prior to BRAF inhibitor treatment (dabrafenib) associated with decreased PFS. | Vergani *et al.* (2011) [17]; Smalley *et al.* (2008) [23]; Nathanson *et al.* (2013) [24]/FoundationOne [6]; KEGG_pathwaysInCancer [5]; LyndaChin-Cell2012 [7]; PanCancer_12Cancer_SignifMutatedGenes_Nature2013 [8]; SaturationAnalysisOn21CancerTypes [9] |
|  | *CCNA1* | X |  | -Cell cycle activation mediates resistance to BRAF inhibitors. | KEGG_pathwaysInCancer [5] |
|  | *CCND2* |  | X | -Gene related to CCND1/Cell cycle activation mediates resistance to BRAF inhibitors. | FoundationOne [6]; KEGG_pathwaysInCancer [5] |
|  | *CCND3* |  | X | -Gene related to CCND1/Cell cycle activation mediates resistance to BRAF inhibitors. | FoundationOne [6]; KEGG_pathwaysInCancer [5] |
|  | *CDKN2A* | X | X | -CDKN2A loss reported in progressive tumors of melanoma patients treated with BRAF inhibitors.  -Lower copy number of CDKN2A in melanoma tumors prior to BRAF inhibitor treatment (dabrafenib) associated with decreased PFS. | Shi *et al.* (2014) [3]; Nathanson *et al.* (2013) [24]/KEGG_pathwaysInCancer [5]; FoundationOne [6]; PanCancer_12Cancer_SignifMutatedGenes_Nature2013 [8]; SaturationAnalysisOn21CancerTypes [9] |
|  | *CDKN1A* | X |  | -Gene related to CDKN2A/Cell cycle activation mediates resistance to BRAF inhibitors. | Shi *et al.* (2014) [3]/KEGG_pathwaysInCancer [5];  PanCancer_12Cancer_SignifMutatedGenes_Nature2013 [8];  SaturationAnalysisOn21CancerTypes [9] |
|  | *CDKN1B* | X |  | -Gene related to CDKN2A/Cell cycle activation mediates resistance to BRAF inhibitors. | Shi *et al.* (2014) [3]/KEGG_pathwaysInCancer [5]; FoundationOne [6]; PanCancer_12Cancer_SignifMutatedGenes_Nature2013 [8]; SaturationAnalysisOn21CancerTypes [9] |
| **Apoptosis related genes** | *BCL2* | X |  | -expression of BCL2 (anti-apoptotic factor) increased following BRAF inhibitor treatment in Vemurafenib-resistant cells | Wei *et al.* (2017) [25]/KEGG_pathwaysInCancer [5]; FoundationOne [6] |
|  | *BCL2L1* | X |  | -BCL2A1 is a lineage-specific anti-apoptotic melanoma oncogene that confers resistance to BRAF inhibition.  -BCL2 related gene/anti-apoptotic mediated BRAF inhibitors resistance. | Haq *et al.* (2013) [26]/KEGG_pathwaysInCancer [5] |
|  | *BCL2L11* | X |  | -BCL2L11 (pro-apoptotic factor) overexpression reported to promote sensibility to BRAF inhibitor (PLX4720) in melanoma cell lines.  -BCL2L11 upregulation described in melanoma cell lines resistant to BRAF inhibitors (PLX4720). | Shao and Aplin (2010) [27]; Lai *et al.* (2012) [28]/KEGG_pathwaysInCancer [5] |
|  | *BMF* | X |  | -BMF (pro-apoptotic factor) overexpression reported to promote sensibility to BRAF inhibitor (PLX4720) in melanoma cell lines. | Shao and Aplin (2010) [27]/KEGG_pathwaysInCancer [5] |
|  | *MCL1* | X |  | -Decreased MCL1 (anti-apoptotic factor) expression reported to promote sensibility to BRAF inhibitor (PLX4720) in melanoma cell lines.  - MCL1 downregulation described in melanoma cell lines resistant to BRAF inhibitor (PLX4720). | Shao and Aplin (2010) [27]; Lai *et al.* (2012) [28]/FoundationOne [6] |
|  | *BAD* | X |  | -Gene related to BCL2L11/expression mediates sensitivity to BRAF inhibitors. | KEGG_pathwaysInCancer [5] |
|  | *RRM2* | X |  | -RRM2 inhibition synergize BRAF inhibitor effect on melanoma cell proliferation | Fatkhutdinov *et al.* (2016) [29]/KEGG_p53SignalingPathway [5] |
|  | *PTEN* | X |  | -PTEN inactivation/down-expression reported to promote resistance to BRAF inhibitors in melanoma cell lines.  -PTEN mutation/down-expression associated with shorter PFS/lower response rate in melanoma patients treated with a BRAF inhibitor. | Paraiso *et al.* (2011) [30]; Nathanson *et al.* (2013) [24]; Shao and Aplin (2010) [27]; Trunzer *et al.* (2013) [31]/FoundationOne [6]; KEGG_pathwaysInCancer [5]; LyndaChin-Cell2012 [7]; PanCancer_12Cancer_SignifMutatedGenes_Nature2013 [8];  SaturationAnalysisOn21CancerTypes [9] |
